# Construction of Consensus Genetic Map With Applications in Gene Mapping of Wheat (*Triticum aestivum* L.) Using 90K SNP Array

**DOI:** 10.3389/fpls.2021.727077

**Published:** 2021-08-25

**Authors:** Pingping Qu, Jiankang Wang, Weie Wen, Fengmei Gao, Jindong Liu, Xianchun Xia, Huiru Peng, Luyan Zhang

**Affiliations:** ^1^The National Key Facility for Crop Gene Resources and Genetic Improvement, Institute of Crop Sciences, Chinese Academy of Agricultural Sciences, Beijing, China; ^2^State Key Laboratory of Agrobiotechnology, Key Laboratory of Crop Heterosis and Utilization, Beijing Key Laboratory of Crop Genetic Improvement, College of Agronomy and Biotechnology, China Agricultural University, Beijing, China; ^3^Department of Cell Biology, Zunyi Medical University, Zunyi, China; ^4^Crop Research Institute, Heilongjiang Academy of Agricultural Sciences, Harbin, China

**Keywords:** wheat (*Triticum aestivum* L.), consensus genetic map, QTL mapping, plant height, spike length, thousand-kernel weight

## Abstract

Wheat is one of the most important cereal crops worldwide. A consensus map combines genetic information from multiple populations, providing an effective alternative to improve the genome coverage and marker density. In this study, we constructed a consensus map from three populations of recombinant inbred lines (RILs) of wheat using a 90K single nucleotide polymorphism (SNP) array. Phenotypic data on plant height (PH), spike length (SL), and thousand-kernel weight (TKW) was collected in six, four, and four environments in the three populations, and then used for quantitative trait locus (QTL) mapping. The mapping results obtained using the constructed consensus map were compared with previous results obtained using individual maps and previous studies on other populations. A simulation experiment was also conducted to assess the performance of QTL mapping with the consensus map. The constructed consensus map from the three populations spanned 4558.55 cM in length, with 25,667 SNPs, having high collinearity with physical map and individual maps. Based on the consensus map, 21, 27, and 19 stable QTLs were identified for PH, SL, and TKW, much more than those detected with individual maps. Four PH QTLs and six SL QTLs were likely to be novel. A putative gene called *TraesCS4D02G076400* encoding gibberellin-regulated protein was identified to be the candidate gene for one major PH QTL located on 4DS, which may enrich genetic resources in wheat semi-dwarfing breeding. The simulation results indicated that the length of the confidence interval and standard errors of the QTLs detected using the consensus map were much smaller than those detected using individual maps. The consensus map constructed in this study provides the underlying genetic information for systematic mapping, comparison, and clustering of QTL, and gene discovery in wheat genetic study. The QTLs detected in this study had stable effects across environments and can be used to improve the wide adaptation of wheat cultivars through marker-assisted breeding.

## Introduction

Wheat (*Triticum aestivum* L.) is one of the most important cereal crops worldwide, providing about one-fifth of the total calories consumed by humans. Due to limited farmland and the rapid increase in human population, there is an urgent need to accelerate the genetic gain on grain yield through advanced genetic research and breeding activities in wheat. Genetic linkage map construction and quantitative trait locus (QTL) mapping are important areas in genetic research, as they provide fundamental information for gene cloning, marker-assisted breeding, and genome structure studies (Meng et al., 2015; Rasheed et al., 2016).

Linkage mapping approach based on individual populations has become routine in wheat genetic studies to dissect the genetic architecture of complex traits. However, a large number of co-localized markers and low marker density due to a limited genetic variation and a limited number of crossing-over events are commonly seen with linkage maps constructed in individual populations. Detected QTLs are usually specific to designated crosses with wide confidence intervals, hindering further genetic research on gene fine-mapping and cloning. Furthermore, linkage mapping in single populations can only identify QTLs with phenotypic variations from specific crosses, and each mapping population can only represent a small number of crossing-over events (Liu and Zeng, 2000). The narrow genetic basis associated with individual crosses and populations reduces both phenotypic and genotypic diversity. One way to solve these problems is to construct a consensus map as the connection across multiple populations.

A consensus genetic map combines genetic information from multiple populations, and therefore provides an effective alternative to improve genome coverage and marker density (Maccaferri et al., 2015; Allen et al., 2017). A higher marker density of the consensus map offers the chance to map more QTLs to narrower intervals and to identify more closely linked markers for the discovery of causal genes and marker-assisted selection (MAS) in breeding. Consensus maps can also be used to validate marker order, characterize genomic diversity, increase the power of genome-wide association studies, and conduct QTL meta-analysis (Cavanagh et al., 2013; Wang et al., 2014; Wingen et al., 2017; Liu et al., 2020).

Some computer tools that can be used for consensus map construction have been developed in the last 20 years, such as BioMercator (Arcade et al., 2004), JoinMap (Van Ooijen, 2006), MergeMap (Wu et al., 2010), MultiPoint (Ronin et al., 2012), and LPmerge (Endelman and Plomion, 2014). Using these tools, consensus maps have been developed for wheat. Somers et al. (2004) reported the first consensus map for wheat based on SSR markers from three doubled haploid (DH) and a recombinant inbred line (RIL) populations. Cavanagh et al. (2013) generated a high-density consensus map from seven populations, consisting of 7,504 single nucleotide polymorphism (SNP) markers. Wang et al. (2014) integrated six bi-parental DH populations to generate a consensus map using 40,267 markers. Liu et al. (2020) developed a consensus map with a total length of 4,080.5 cM containing 47,309 markers based on 21 individual linkage maps and three previously reported consensus maps.

In this study, a consensus genetic map was constructed using three bi-parental populations of RILs in wheat. QTL mapping was then conducted for plant height (PH), spike length (SL), and thousand-kernel weight (TKW) using the constructed consensus map. The mapping results were compared among populations, and with the results obtained using individual maps with the purpose of identifying stable and common QTLs. In addition, a simulation experiment was conducted to demonstrate the advantages of using a consensus map in QTL mapping.

## Materials and Methods

### Plant Materials and Phenotyping Experimental Design

The three recombinant inbred line populations used in this study were derived from crosses Doumai × Shi 4185 (denoted as DS, 275 F_2:6_ RILs), Gaocheng 8901 × Zhoumai 16 (denoted as GZ, 176 F_2:6_ RILs), and Zhou 8425B × Chinese Spring (denoted as ZC, 245 F_2:8_ RILs), which had been previously reported by Wen et al. (2017). Population DS and its parental lines were planted at Shunyi (Beijing, China) and Shijiazhuang (Hebei, China) in 2012–2013, 2013–2014, and 2014–2015 cropping seasons. Population GZ and its parental lines were planted at Anyang (Henan, China) and Suixi (Anhui, China) in 2012–2013 and 2013–2014 cropping seasons. Population ZC and its parental lines were planted at Zhengzhou and Zhoukou (Henan, China) in 2012–2013 and 2013–2014 cropping seasons. Randomized complete block designs with three replications were used in field trials. Each plot had three rows with 1.5 m in length and 0.2 m apart between rows. About 50 seeds were sown in each row. Field management was performed according to local practices.

Plant height was recorded as the average height based on 10 representative plants, measured from the base of the stem to the top of the spike excluding awns at the late grain-filling stage. SL was recorded as the average length of 20 representative spikes in populations DS and GZ, and five representative spikes in population ZC, measured from the base of the spike to the top of the spike excluding awns. TKW was evaluated by weighing three random samples of 500 kernels from each plot after harvest.

### Genotyping and Marker Quality Control

Deoxyribonucleic acid was extracted from leaves of 15-day-old seedlings according to the cetyltrimethyl ammonium bromide (CTAB) protocol (Sharp et al., 1988). The populations were genotyped by the 90K wheat Infinium iSelect SNP array (Wang et al., 2014) at CapitalBio Corporation (http://www.capitalbio.com) in Beijing, China. Quality control of the genotypic data has been previously described in Wen et al. (2017), and described here briefly and as follows. First, heterozygous marker types were set as missing values. Then, markers with more than 10% of missing values were deleted. Finally, SNPs with minor allelic frequency lower than 0.3 were filtered out. The three individual linkage maps based on these markers were reported by Wen et al. (2017). SNPs on the three maps were used for consensus map construction. The R package VennDiagram (Chen and Boutros, 2011) was used to demonstrate the SNP numbers common among the three individual maps.

### Statistical Analysis for Phenotypic Data

Analysis of variance and calculation of broad-sense heritability (*H*^2^) from phenotypic data were performed using the AOV function in software QTL IciMapping V4.2 (Meng et al., 2015). Pearson correlation coefficients among traits were calculated using mean phenotypic values across environments.

### Consensus Genetic Map Construction

First, markers from the three recombinant inbred line populations were grouped according to their chromosome information in individual maps reported by Wen et al. (2017). Markers that were present on the same chromosome in the three individual maps were treated as anchors. Then, an algorithm called combined linkage analysis (CLA, developed by the group of the authors) was used for consensus map construction. To assure the quality of the map, a limited number of markers were removed manually if they caused serious inconsistency in the marker order between the genetic and physical maps, or excessive expansion of the constructed genetic map. The R package LinkageMapView (Ouellette et al., 2018) was used to visualize the constructed consensus map.

Furthermore, four steps were involved in the CLA algorithm: step 1: derive the theoretical recombination frequencies of pairwise markers in each mapping population; step 2: estimate the recombination frequency between two linked markers and sampling variance of the estimated recombination frequency in each population. In addition to RIL populations, CLA is applicable to many other kinds of bi-parental populations, as described in Meng et al. (2015). For some kinds of mapping populations such as DH and RIL, the likelihood equation on recombination frequency has an explicit solution, so the maximum likelihood estimate can be calculated directly. For other kinds of mapping populations such as F_2_ and F_3_, the maximum likelihood estimate cannot be succinctly given. In this situation, either Newton iteration or the expectation-maximization (EM) algorithm has to be adopted in estimating the recombination frequency (Zhang et al., 2019). Step 3: estimate the combined recombination frequency using the estimates and their sampling variances from individual populations; reciprocal of sampling variance of the estimated recombination frequency is used as the weight of the corresponding population. Weight is set as zero for those populations where the pair-wise recombination frequency cannot be estimated. Step 4: construct the consensus linkage map based on the combined estimates of recombination frequencies between markers; a combination of the nearest-neighbor algorithm and a two-opt algorithm in solving the traveling salesman problem (TSP) was used in the marker ordering (Zhang et al., 2020a).

### Comparison of Marker Orders in the Consensus Map, Physical Map, and Individual Genetic Maps

Spearman rank correlation was used to measure the collinearity of marker orders between the different maps, which was calculated by the R Software. Marker order in each chromosome in the consensus map was compared with the physical map order of the respective chromosome. To acquire the physical positions of the markers, sequences of SNPs were used to BLAST (Basic Local Alignment Search Tool) against the wheat genome IWGSC RefSeq v2.0 (https://urgi.versailles.inra.fr/download/iwgsc/IWGSC_RefSeq_Assemblies/v2.0/, International Wheat Genome Sequencing Consortium). The E-value threshold in BLAST was set at 10^−10^. The markers were filtered out if their alignment lengths were lower than 80% of the query sequence length or the identities were lower than 0.85. If a marker was assigned to multiple chromosomes by BLAST, the position on the same chromosome as the consensus map was used in collinearity analysis. Marker order comparison was also conducted between the consensus map and individual maps, as well as among the three individual maps. For each comparison, only the common markers on two maps were used in the calculation of collinearity.

### QTL Mapping Based on the Consensus Map

Quantitative trait locus mapping was conducted in the individual populations using the consensus map. The inclusive composite interval mapping (ICIM) implemented in the BIP function in QTL IciMapping V4.2 (Meng et al., 2015) was applied on the mean phenotypic values across blocks in each environment and best linear unbiased estimation (BLUE) values across multiple environments. Scanning step was set at 0.2 cM. Probabilities of adding and removing variables in stepwise regression were set at 0.001 and 0.002, respectively. Threshold logarithm of odd (LOD) score was set at 2.5, same as the QTL mapping studies on individual maps from the three populations (Gao et al., 2015; Li et al., 2018).

Quantitative trait loci and quantitative trait locus clusters were named with chromosomal locations, considering all populations together. QTLs detected in the same population were considered to be common if the distance between QTL positions was <20 cM in different environments. QTLs detected in different populations were considered to be common if the genetic and physical positions were close enough. In other words, distance in the linkage map was <20 cM in terms of QTL positions, and distance in the physical map was <25 Mb in terms of the minimum physical distances between flanking makers. In individual populations, QTLs are considered to be stable if they are identified in at least half of tested environments. Stable QTLs for different traits were classified into the same cluster if the minimum distance between the QTL confidence intervals was <15 cM. The shiny Circos tool (Yu et al., 2018) was used to visualize QTL positions on the consensus map. Stable QTLs detected with the consensus map in this study were compared with those detected by ICIM using individual maps (Gao et al., 2015; Li et al., 2018), according to physical and genetic positions of the flanking markers.

### Genetic Models Used in Simulation

A simulation study was conducted to compare the QTL mapping results from the individual and consensus maps. We assumed that a chromosome has a length of 100 cM and contains 101 evenly distributed markers. Considering that the consensus map always has more markers than each individual map, we assume that the consensus map contained all the 101 markers, but that the individual map only contained half of them, i.e., 51 evenly distributed markers with marker density at 2 cM. Three QTL distribution models were simulated (Supplementary Table 1). In model I, a QTL was located at 34.5 cM on the chromosome with an additive effect of 1. In model II, two QTLs were linked in the coupling phase, both with an additive effect of 1. In model III, two QTLs were linked in the repulsion phase with additive effects of −1 and 1, respectively. The broad sense heritability (*H*^2^) was set at three levels, i.e., 0.05, 0.1, and 0.2 for model I, and 0.1, 0.2, and 0.4 for models II and III. One thousand RIL populations, each with a size of 200, were simulated for each model, and each heritability level by the BIP simulation functionality was implemented in QTL Ici Mapping V4.2 (Meng et al., 2015). The consensus map with 101 markers and the predefined QTLs were used to generate the simulated populations. Both the consensus and individual maps were used in QTL mapping. For QTL mapping using individual maps, genotypic data of the 51 markers were used. For QTL mapping using the consensus map, genotypic data of the 51 markers were the same as those in individual maps, but the other 50 markers only present in the consensus map were set as missing values. For the ICIM QTL mapping method on simulated populations, the scanning step was set at 0.1 cM and the threshold LOD score was set at 2.5. Probabilities for entering and removing variables in the stepwise regression were set at 0.001 and 0.002, respectively. QTL detection power was estimated according to a support interval of 5 cM centered at the position of true QTL. If multiple peaks occurred within the support interval, only the highest one was counted. QTLs identified out of the support interval were regarded as false positives (Li et al., 2012). The other parameters were set as default values.

## Results

### General Information on Both Genotypic and Phenotypic Data

There were 10,986 markers on the linkage map constructed in population DS, 11,819 markers in population GZ, and 14,862 markers in population ZC. Populations DS and GZ shared 4,208 common markers; DS and ZC shared 4,420 common markers; GZ and ZC shared 5,183 common markers; the three populations had 1,880 markers in common (Supplementary Figure 1). A total of 25,736 unique markers on the three individual maps were used for consensus map construction.

Phenotypic means and heritability of the three traits are shown in Table 1 for the three RIL populations across a number of environments. Frequency distributions in different populations and environments are shown in Supplementary Figure 2 for PH, Supplementary Figure 3 for SL and Supplementary Figure 4 for TKW. For PH, Doumai was taller than Shi 4185 in four environments, but shorter in the other two environments in population DS; Gaocheng8901 was always taller than Zhoumai 16 in population GZ; Chinese Spring was always taller than Zhou 8425B in population ZC (Supplementary Figure 2). For SL, Doumai was longer than Shi 4185 in four environments, almost equal in one environment, and shorter in the other one environment; Zhoumai16 was longer than Gaocheng 8901 in three environments, and shorter in the other environment; Chinese Spring was always longer than Zhou 8425B (Supplementary Figure 3). For TKW, Doumai was always higher than Shi 4185; Zhoumai 16 was always higher than Gaocheng 8901; Zhou 8425B was always higher than Chinese Spring (Supplementary Figure 4). The three traits were continuously distributed in the three populations, similar to and typical in most QTL mapping studies. Much wider ranges were observed in the progenies in comparison with their parents, except for TKW in two environments in population ZC (Supplementary Figures 2–4). Heritability in the broad sense, based on the replicated means, was quite high for the three traits, ranging from 0.91 to 0.99 (Table 1), while heritability based on the plot level ranged from 0.59 to 0.91. Correlation coefficients between traits in the three populations are given in Supplementary Table 2. At a significance level of 0.01, PH was positively correlated with TKW in all three populations. SL showed a positive correlation with both PH and TKW in population DS. Other correlations were non-significant.

**Table 1:** Mean performance and heritability of plant height (PH), spike length (SL), and thousand-kernel weight (TKW) in the three RIL populations, Doumai × Shi 4185 (DS), Gaocheng 8901 × Zhoumai 16 (GZ), and Zhou 8425B × Chinese Spring (ZC), across multiple environments.

**Population**	**Trait**	**Parent^a^**		**RIL population^b^**			***H*^**2**^_by_mean^c^**	***H*^**2**^_by_plot^d^**
		**P1**	**P2**	**Mean**	**SD**	**Range**		
DS	PH	73.51	73.54	83.89	7.56	64.99–105.09	0.97	0.69
	SL	9.29	8.30	8.77	0.97	6.17–12.10	0.95	0.62
	TKW	50.30	35.35	43.56	4.95	30.52–60.10	0.96	0.75
GZ	PH	94.38	67.77	90.67	15.82	44.23–118.25	0.99	0.91
	SL	8.74	8.97	8.59	0.87	6.60–11.24	0.96	0.72
	TKW	43.83	48.17	46.52	3.81	33.46–55.55	0.91	0.59
ZC	PH	67.12	115.08	101.07	14.02	60.58–125.87	0.95	0.83
	SL	11.50	8.31	10.14	1.15	6.89–13.83	0.91	0.72
	TKW	52.63	29.10	37.12	4.16	26.52–48.83	0.94	0.81

a *Best linear unbiased estimation (BLUE) values across multiple environments. In population DS, P1 and P2 refer to Doumai and Shi 4185, respectively. In population GZ, P1 and P2 refer to Gaocheng 8901 and Zhoumai 16, respectively. In population ZC, P1 and P2 refer to Zhou 8425B and Chinese Spring, respectively*.

b *Values were based on BLUE across multiple environments*.

c *Heritability in broad sense based on replicated means*.

d *Heritability in broad sense based on plot level*.

*SD, standard deviation*.

### Characteristics of the Constructed Consensus Map

**Figure 1:**
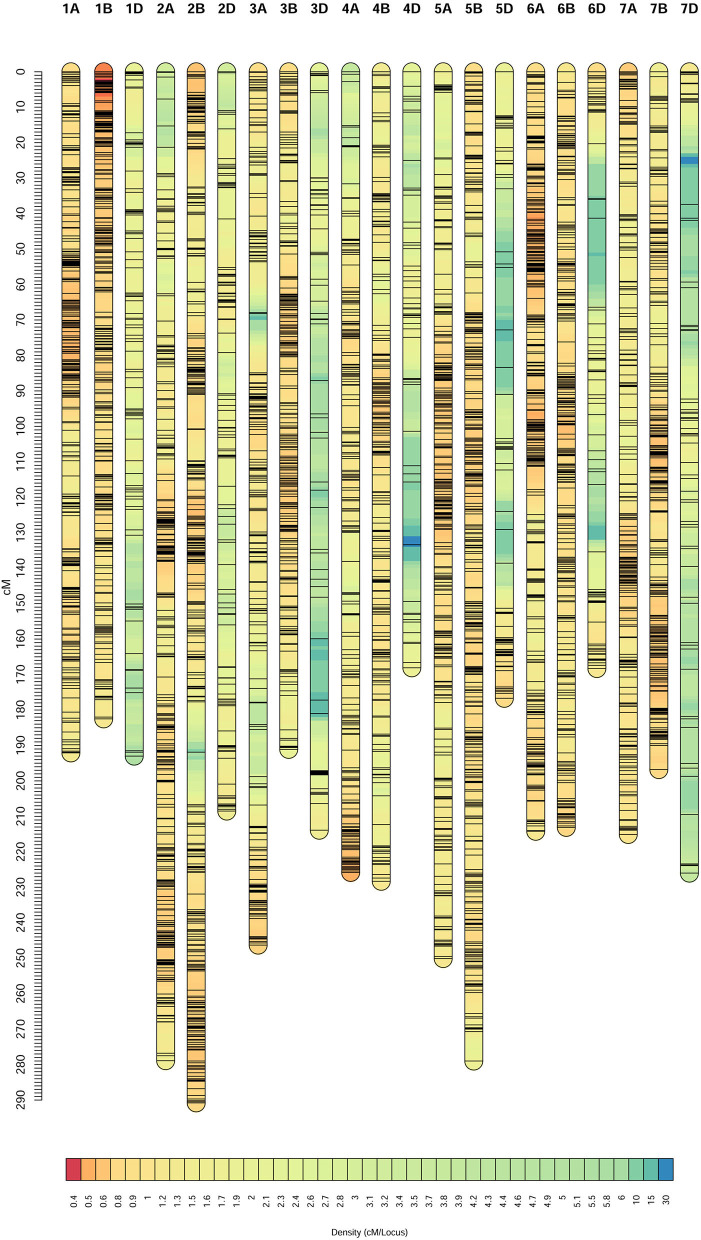
Consensus genetic map constructed from the three recombinant inbred line (RIL) populations, Doumai × Shi 4185 (DS), Gaocheng 8901 × Zhoumai 16 (GZ), and Zhou 8425B × Chinese Spring (ZC).

Of the 25,736 unique SNPs on the three individual linkage maps, 25,667 were assigned to the consensus map, resulting in 21 linkage groups corresponding to the 21 chromosomes in hexaploid wheat (Figure 1). General information on the consensus map is provided in Table 2, and positions of all the markers on both the genetic and physical maps are given in Supplementary Table 3. The consensus map spanned 4,558.55 cM in length, and the number of unique map positions (denoted as bins) was equal to 3,979. Lengths of the A, B, and D genomes were 1,622.47, 1,581.57, and 1,354.52 cM, respectively (Table 2). Chromosome 4D was the shortest, with a length of 168.06 cM, and had the least number of markers (i.e., 106) and the least number of bins (i.e., 53). Chromosome 2B was the longest with a length of 290.78 cM, and had the second largest number of markers (i.e. 2,431) and the second largest number of bins (i.e., 324). There were 18 gaps longer than 15 cM on the consensus map, 16 of which were located in the D genome (Supplementary Table 3). Average distance between adjacent bins was equal to 1.15 cM.

**Table 2:** Characteristics of the consensus genetic map constructed from the three RIL populations, DS, GZ, and ZC.

**Chromosome**	**Length (cM)**	**Marker number**	**Bin number**	**Average BD (cM)^a^**	**Max BD (cM)^b^**	**Coefficient^c^**	**Consistent proportion (%)^d^**
1A	192.09	1280	240	0.80	9.22	0.99	50.55
1B	182.47	2014	257	0.71	5.72	0.95	51.07
1D	192.98	700	82	2.35	19.42	0.96	57.93
2A	278.86	1685	268	1.04	10.08	0.99	51.99
2B	290.78	2431	324	0.90	14.73	0.87	45.67
2D	208.48	625	108	1.93	13.68	0.99	71.76
3A	246.30	1381	195	1.26	16.68	0.98	55.72
3B	191.12	1974	219	0.87	9.66	0.94	43.30
3D	213.91	294	60	3.57	23.97	0.74	46.04
4A	225.78	1304	193	1.17	12.36	0.98	47.22
4B	228.26	713	201	1.14	8.88	0.98	58.53
4D	168.06	106	53	3.17	17.71	0.95	75.68
5A	250.07	1238	255	0.98	18.50	0.96	62.41
5B	279.03	2475	330	0.85	8.33	0.95	46.34
5D	176.72	298	67	2.64	17.76	0.96	68.11
6A	214.24	1696	289	0.74	7.26	0.96	36.30
6B	213.08	1571	252	0.85	6.04	0.96	53.88
6D	168.33	350	73	2.31	25.08	0.96	54.44
7A	215.12	1701	213	1.01	8.00	0.99	62.07
7B	196.83	1577	236	0.83	7.77	0.98	45.75
7D	226.05	254	64	3.53	28.84	0.99	73.71
**Genome**							
A	1622.47	10285	1653	0.98	18.50	0.98	52.32
B	1581.57	12755	1819	0.87	14.73	0.98	49.22
D	1354.52	2627	507	2.67	28.84	0.94	63.95
**Homeologous groups**							
1	567.54	3994	579	0.98	19.42	0.97	53.19
2	778.12	4741	700	1.11	14.73	0.95	56.47
3	651.33	3649	474	1.37	23.97	0.89	48.35
4	622.10	2123	447	1.39	17.71	0.97	60.48
5	705.82	4011	652	1.08	18.50	0.95	58.95
6	595.64	3617	614	0.97	25.08	0.96	48.21
7	637.99	3532	513	1.24	28.84	0.99	60.51
Total	4558.55	25667	3979	1.15	28.84	0.95	55.17

a *Average distance between two adjacent bins*.

b *Maximum distance between two adjacent bins*.

c *Spearman rank correlation coefficient between the consensus map and IWGSC RefSeq v2.0*.

d *The proportion of SNPs arranged in the order same with those on the corresponding chromosomes of the physical map*.

The single nucleotide polymorphism markers (SNPs) number was similar in the A and B genomes, i.e., 10,285 and 12,755 SNPs, but the number was much lower in the D genome, i.e., 2,627 SNPs (Table 2). In comparison with the A and B genomes, the D genome was shorter and contained much fewer markers and bins, and more gaps, indicating that fewer crossing-over events happened on the D genome, which was also observed in the three individual maps. Although the marker number and bin number in the D genome were significantly lower than those in the A and B genomes, results from BLAST indicated that the constructed consensus map still had nearly complete coverage for chromosomes in the D genome.

**Figure 2:**
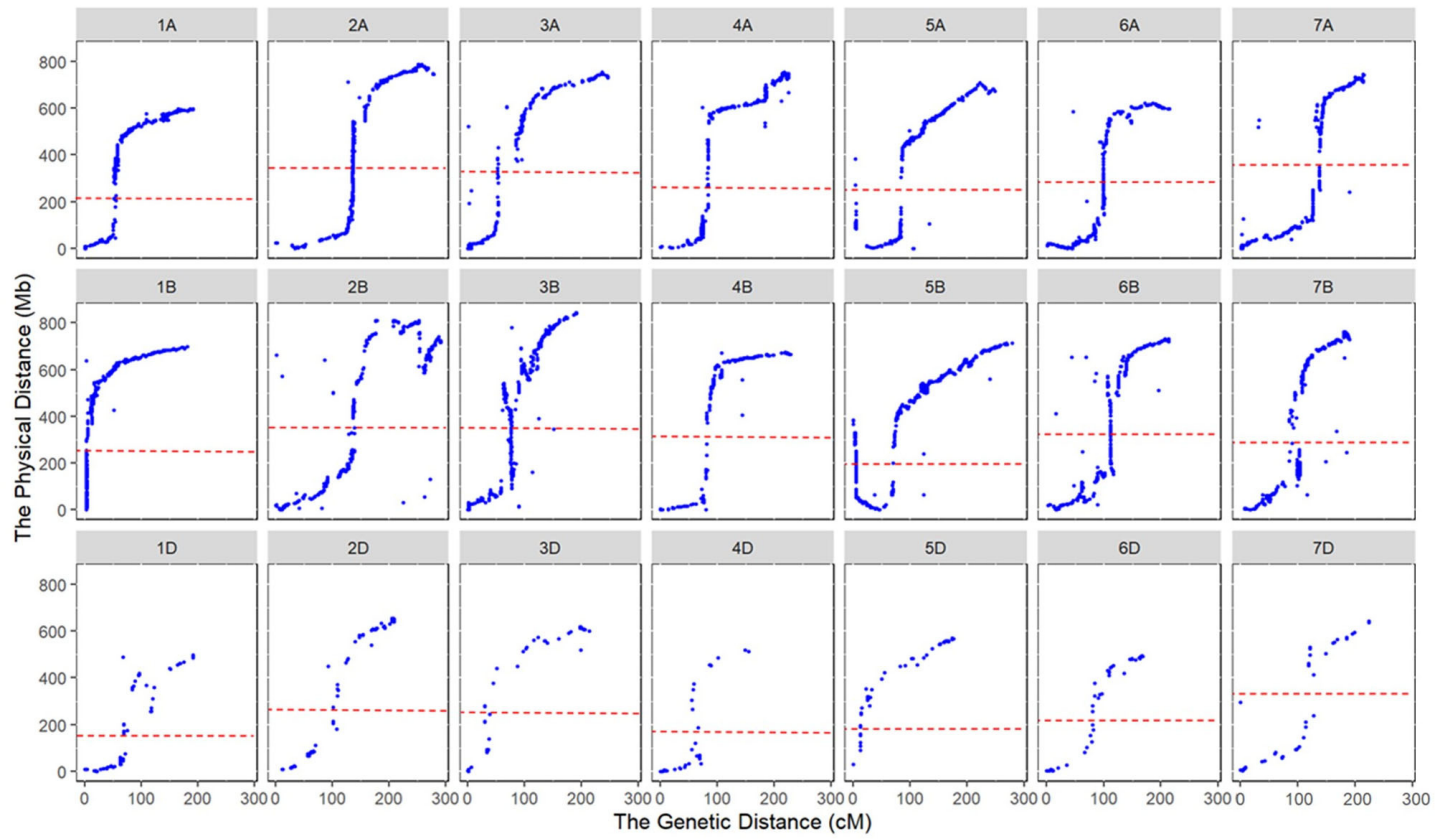
Collinearity of marker orders between the consensus and physical maps. The dotted lines indicate the centromeres of chromosomes.

Marker orders on the consensus map and physical map had high collinearity, with an average Spearman rank correlation coefficient of 0.95 across the 21 chromosomes (Table 2, Figure 2). Rank correlation coefficients were higher than 0.94 for all the chromosomes except 2B and 3D. The lower coefficient observed on 3D may be partly due to the much-reduced bin number when many markers were clustered in bins. Collinearity analysis between the consensus and physical maps also revealed that markers in large physical region around the centromeres of chromosomes tended to be clustered in a short genetic interval on consensus genetic map (Figure 2), indicating a much stronger recombination suppression occurred around the centromere than did that the distal regions.

### Comparison of the Consensus Map With the Three Individual Maps

Wen et al. (2017) reported three linkage maps from three populations constructed with QTL IciMapping V4.0 (Meng et al., 2015), JoinMap 4.0 (Stam, 1993), and MapDisto 1.7 (Lorieux, 2012). Two of them had 21 linkage groups, and one had 31 linkage groups. The consensus map constructed in this study had 21 linkage groups, corresponding to the 21 chromosomes in hexaploid wheat. The marker and bin numbers on the consensus map were 1.73 and 1.15 times higher than the largest marker and bin numbers on the three individual maps. The length of the consensus map was 1.44 times longer than that of the longest individual map. Longer chromosomes on the individual maps also tended to be longer on the consensus map. For example, the two longest chromosomes on the consensus map, i.e., 2B and 5B, ranked first and third in mapping length in each of the three individual maps.

There were 616 markers with inconsistent chromosomes on the individual maps, but the inconsistent chromosomes for each marker were finalized to one unique chromosome on the consensus map (Supplementary Table 4). Among these markers, 540 were mapped to single chromosomes that they were located on the individual maps. For example, marker wsnp_Ex_c200_391015 was located on chromosomes 7A and 1A on individual maps of populations GZ and ZC, respectively, which was finalized on chromosome 1A on the consensus map. Forty-nine markers were mapped to one of the homeologous chromosomes. For example, marker Tdurum_contig28665_150 was located on chromosomes 1D, 1D, and 2A in populations DS, GZ, and ZC, respectively, and was finalized on chromosome 1A, a homeologous chromosome of 1D. Twenty-seven markers were mapped to neither the same chromosome nor homeologous chromosomes. For example, marker tplb0024a09_2369 was located on chromosomes 7D and 4A in populations DS and ZC, respectively, and was finalized on chromosome 2B on the consensus map (Supplementary Table 4).

The markers showed high collinearity across chromosomes between the consensus and individual maps, and the average Spearman rank correlation coefficient was similar to those between the individual maps (Supplementary Table 5). Fewer inconsistencies in orders between the consensus and individual maps were observed for closely linked markers.

### QTLs for PH Detected From the Consensus Map and Comparison With Those From Individual Maps

**Figure 3:**
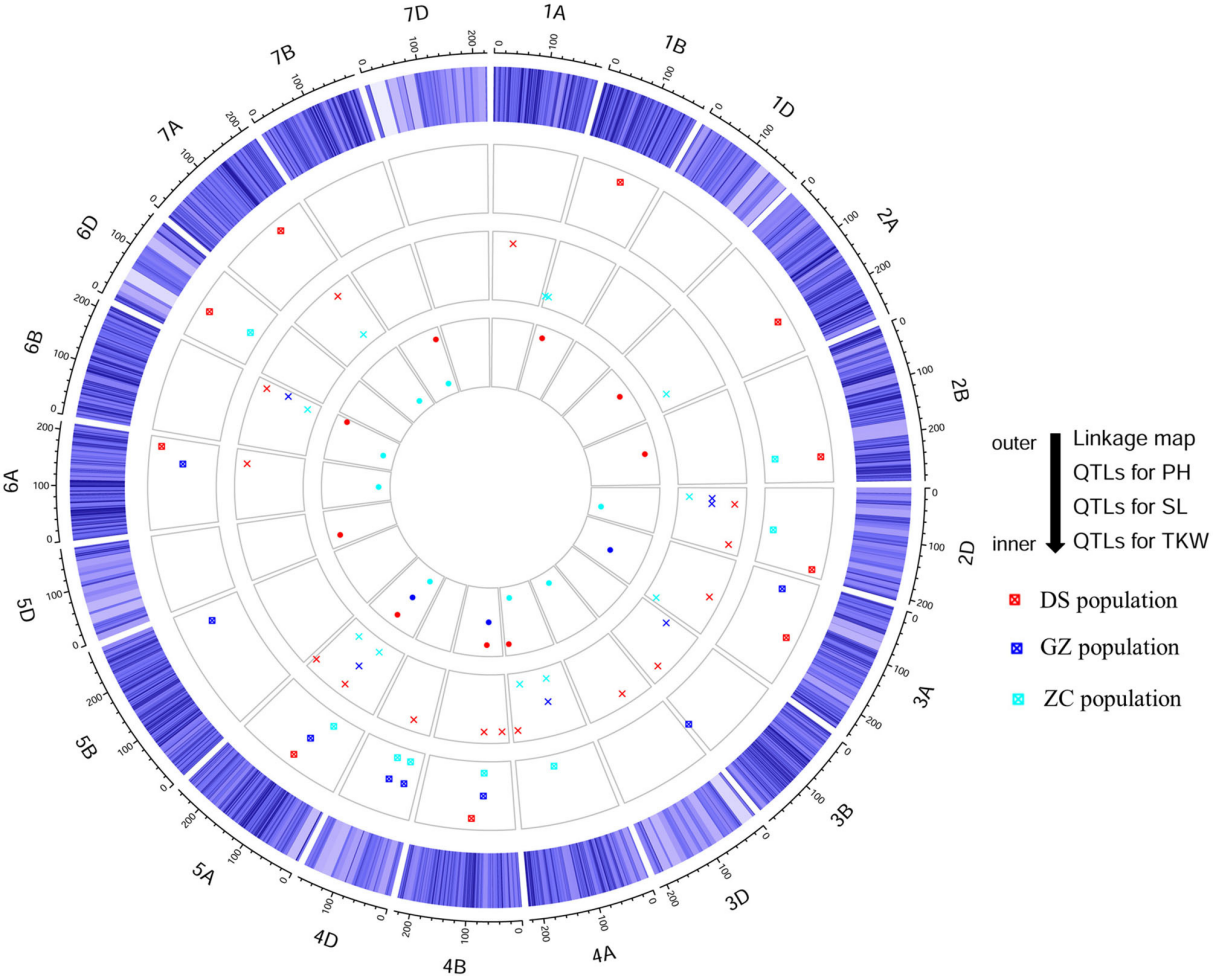
Schematic representation of stable quantitative trait loci (QTLs) for plant height (PH), spike length (SL), and thousand-kernel weight (TKW) detected in three RIL populations, DS, GZ, and ZC from the consensus map.

Using the consensus map, a total of 40 QTLs were detected for PH (Supplementary Table 6), among which 10, 8, and 8 were stable in populations DS, GZ, and ZC, respectively (Figure 3, Table 3). Five QTLs were identified in two populations, i.e., *qPH-2B-2, qPH-4B-1, qPH-4D-1, qPH-4D-2*, and *qPH-5A-2*. *qPH-2B-2* were repeatedly detected in populations DS and ZC with LOD scores in the range of 3.62 to 22.98, explaining 1.63–8.05% of the phenotypic variance (PVE). *qPH-5A-2* was repeatedly detected in populations DS and GZ, with LOD scores ranging from 3.90 to 15.44, and PVE values ranging from 2.58 to 9.63%. *qPH-4B-1, qPH-4D-1*, and *qPH-4D-2* were repeatedly identified in populations GZ and ZC, taking the top three ranks in both populations by average LOD score, PVE value, and additive effect across environments. *qPH-4B-1* was mapped on chromosome 4B at the interval of 34.98–49.79 Mb on physical map with LOD scores ranging from 6.31 to 43.49, and PVE values ranging from 8.14 to 30.85%. *qPH-4D-1* was mapped on chromosome 4D at the interval of 14.14–17.01 Mb with LOD scores ranging from 6.54 and 17.10, and PVE values ranging from 8.06 to 16.48%. *qPH-4D-2* was mapped on chromosome 4D at the interval of 32.97–65.01 Mb having LOD scores ranging from 4.09 to 15.72 and PVE values ranging from 5.03 to 12.03%. When the length of the confidence interval was set at 25 Mb, *qPH-4B-1* and *qPH-4D-1* were, respectively, coincident with dwarfing genes *Rht-B1* located at 33.61 Mb on 4B and *Rht-D1* located at 19.19 Mb on 4D (IWGSC RefSeq v2.0).

**Table 3:** Stable quantitative trait loci (QTLs) identified for PH in the three RIL populations, DS, GZ, and ZC using the consensus map.

**QTL**	**Pop**	**Environments**	**Position (cM)**	**LOD**	**PVE (%)**	**Add**
*qPH-1B-2*	DS	E2/E3/E4/E5/B	95.60–100.20	3.26–38.07	1.18–15.43	1.37 to 5.42
*qPH-2A-2*	DS	E1/E3/E4/E6/B	192.00–195.20	2.79–11.05	1.01–5.32	−2.28 to −1.15
*qPH-2B-2*	DS	E1/E2/E4/E5/B	224.40–239.60	3.62–22.98	1.63–8.05	−3.92 to −1.26
	ZC	E11/E13/E14/B	225.80–227.80	3.89–5.52	2.33–4.30	−3.20 to −2.31
*qPH-2D-1*	ZC	E13/E14/B	113.80–114.40	2.89–3.73	1.49–3.08	2.01 to 2.77
*qPH-2D-3*	DS	E1/E2/E3/E4/B	190.20–193.60	2.58–17.02	0.75–7.21	1.21 to 3.22
*qPH-3A-1*	GZ	E7/E8/E9/E10/B	34.20–36.80	2.85–4.00	3.48–5.41	2.74 to 4.47
*qPH-3A-2*	DS	E1/E2/E3/E5/B	130.40–148.60	16.23–43.54	6.67–28.31	−5.25 to −3.25
*qPH-3B-2*	GZ	E7/E10/B	190.60–190.60	2.85–3.33	3.53–4.06	3.08 to 3.77
*qPH-4A-2*	ZC	E11/E13/E14/B	114.60–132.80	3.27–4.43	1.33–4.47	2.36 to 2.60
*qPH-4B-1*	GZ	E7/E8/E9/E10/B	75.00–75.00	6.31–9.52	8.14–10.82	−5.72 to −3.92
	ZC	E11/E12/E13/E14/B	74.20–74.40	16.87–43.49	17.92–30.85	5.37 to 11.02
*qPH-4B-2*	DS	E2/E3/E4/E5/E6/B	100.40–102.40	3.12–5.91	1.64–2.54	−1.89 to −1.34
*qPH-4D-1*	GZ	E7/E8/E9/E10/B	33.20–36.00	6.68–8.86	8.06–13.62	4.71 to 6.87
	ZC	E11/E12/E13/E14/B	33.40–34.60	6.54–17.10	8.64–16.48	3.90 to 5.83
*qPH-4D-2*	GZ	E7/E8/E9/E10/B	73.20–73.80	6.20–7.83	8.39–10.90	4.57 to 5.58
	ZC	E11/E12/E13/E14/B	70.80–70.80	4.09–15.72	5.03–12.03	3.00 to 5.54
*qPH-5A-1*	ZC	E11/E12/B	76.40–86.20	2.60–3.72	2.07–4.60	1.87 to 2.84
*qPH-5A-2*	DS	E1/E2/E3/E4/E5	120.60–125.40	8.34–15.44	2.58–9.63	2.24 to 3.26
	GZ	E7/E8/E9/E10/B	113.20–135.60	3.90–5.52	4.79–6.87	−4.89 to −3.09
*qPH-5B*	GZ	E7/E8/E9/E10/B	234.80–237.40	3.60–4.46	4.09–5.54	2.91 to 4.36
*qPH-6A-1*	GZ	E7/E9/B	154.40–157.00	2.81–3.88	3.36–4.04	−3.35 to −3.03
*qPH-6A-2*	DS	E1/E3/B	192.60–192.60	4.10–9.47	1.45–4.48	−2.09 to −1.26
*qPH-6D-1*	DS	E1/E2/E4/E6/B	71.40–76.20	3.21–6.98	1.33–2.46	1.30 to 2.00
*qPH-6D-2*	ZC	E11/E13/E14/B	84.60–84.60	2.75–5.70	1.89–3.38	1.94 to 2.88
*qPH-7A*	DS	E1/E2/E3/E5/B	142.40–145.00	4.84–6.74	1.12–3.11	1.44 to 1.89

*Pop, population; LOD, logarithm of odd; PVE, percentage of phenotypic variance explained; Add, additive effect; E1, 2012–2013 Beijing; E2, 2012–2013 Shijiazhuang; E3, 2013–2014 Beijing; E4, 2013–2014 Shijiazhuang; E5, 2014–2015 Beijing; E6, 2014–2015 Shijiazhuang; E7, 2012–2013 Anyang; E8, 2012–2013 Suixi; E9, 2013–2014 Anyang; E10, 2013–2014 Suixi; E11, Zhoukou2013; E12, Zhengzhou2013; E13, Zhoukou2014; E14, Zhengzhou2014; B, best linear unbiased estimation*.

Quantitative trait locus mapping using the individual maps identified a total of 19 stable QTLs in the three populations, nine in population DS, and five each in populations GZ and ZC (Gao et al., 2015; Li et al., 2018). Sixteen of them were detected using the consensus map; Fifteen of which were stable across environments (Supplementary Table 7, Supplementary Figure 5). *qPH-2B-2* and *qPH-5A-2* were detected only in one population with the individual maps, but in two populations with the consensus map (Table 3, Supplementary Table 7), indicating the reliability of the two QTLs. With the consensus map, eight other stable QTLs were identified for PH, i.e., *qPH-2D-1, qPH-2D-3, qPH-3B-2, qPH-4D-2, qPH-6A-1, qPH-6A-2, qPH-6D-2*, and *qPH-7A*, three in population DS, two each in populations GZ and ZC, and one in populations GZ and ZC.

### QTLs for SL Detected From the Consensus Map and Comparison With Those From the Individual Maps

Using the consensus map, a total of 54 QTLs were detected for SL (Supplementary Table 6), among which 15, 6, and 11 were stable in populations DS, GZ, and ZC, respectively (Figure 3, Table 4). *qSL-2D-1* was repeatedly identified in populations GZ and ZC with LOD scores ranging from 2.67 to 20.91, and PVE values ranging from 2.85 to 31.06%. *qSL-2D-2* was repeatedly detected in populations DS and GZ with LOD scores ranging from 3.20 to 6.40, and PVE values ranging from 1.60 to 6.68%. *qSL-5A-2* was repeatedly identified in populations DS and GZ with LOD scores ranging from 3.31 to 13.93, and PVE values ranging from 1.87 to 7.13%. *qSL-6B-4* was repeatedly detected in the three populations and mapped at chromosome 6B in the interval of 705.19–707.59 Mb on physical map, accounting for 3.36–21.30% of the phenotypic variance.

**Table 4:** Stable QTLs identified for SL in the three RIL populations, DS, GZ, and ZC using the consensus map.

**QTL**	**Pop**	**Environment**	**Position (cM)**	**LOD**	**PVE (%)**	**Add**
*qSL-1A-1*	DS	E1/E2/E4/E6/B	53.60–75.80	2.96–6.05	1.47–4.43	−0.22 to −0.14
*qSL-1B-1*	ZC	E13/E14	2.60–3.80	4.53–6.20	4.13–5.67	−0.35 to −0.28
*qSL-1B-2*	ZC	E11/B	16.20–16.20	3.33–3.37	2.90–3.88	−0.22 to −0.29
*qSL-2A-1*	ZC	E11/E13/E14/B	210.80–228.60	2.66–6.55	2.34–5.72	−0.33 to −0.20
*qSL-2D-1*	GZ	E7/E8/E9/E10/B	32.00–43.20	11.74–20.91	9.84–31.06	0.36 to 0.59
	ZC	E11/E12/E13/E14/B	29.20–41.60	2.67–10.18	2.85–8.91	−0.41 to −0.24
*qSL-2D-2*	DS	E3/E5/B	50.60–55.00	3.20–5.97	1.60–3.75	−0.18 to −0.14
	GZ	E9/E10/B	55.80–57.00	3.97–6.40	2.82–6.68	0.21 to 0.27
*qSL-2D-3*	DS	E1/E2/E3/E4/E5/B	168.80–192.20	3.35–13.66	2.40–6.17	0.17 to 0.29
*qSL-3A-4*	DS	E2/E5/B	130.40–139.40	3.71–6.00	1.62–2.45	−0.15 to −0.15
*qSL-3A-5*	ZC	E12/E14/B	221.00–238.00	2.76–3.80	2.51–3.06	−0.22 to −0.22
*qSL-3B-2*	GZ	E7/E10	23.60–24.80	3.47–3.80	3.55–4.88	−0.23 to −0.20
*qSL-3B-5*	DS	E2/E4/E6/B	147.60–148.00	4.07–7.80	1.69–5.75	0.16 to 0.26
*qSL-3D-2*	DS	E1/E2/E5/B	85.20–87.00	4.05–6.81	2.50–2.72	−0.20 to −0.16
*qSL-4A-1*	ZC	E11/E12/E13/E14/B	73.00–83.20	3.07–12.13	3.27–11.74	−0.45 to −0.26
*qSL-4A-2*	GZ	E8/E9/E10/B	85.00–103.60	4.20–6.29	2.78–7.71	−0.26 to −0.22
*qSL-4A-3*	ZC	E11/E12/E13/E14/B	178.20–187.00	6.25–12.99	6.42–11.77	0.34 to 0.47
*qSL-4A-4*	DS	E1/E2/E5/B	203.40–215.20	5.00–5.90	2.01–3.34	0.14 to 0.23
*qSL-4B-1*	DS	E1/E2/B	21.00–21.40	4.50–7.41	1.77–3.25	0.14 to 0.23
*qSL-4B-2*	DS	E1/E2/E3/E4/E5/E6/B	75.00–80.00	4.90–31.94	3.60–16.99	0.21 to 0.45
*qSL-4D*	DS	E1/E2/E5/B	56.20–66.60	3.54–16.74	1.55–7.79	0.12 to 0.33
*qSL-5A-1*	ZC	E13/E14	89.80–93.60	2.92–3.83	2.60–3.13	0.21 to 0.24
*qSL-5A-2*	DS	E1/E2/E5/E6/B	124.20–124.60	4.28–13.93	1.87–7.13	0.15 to 0.34
	GZ	E7/E8	122.60–123.40	3.31–4.39	3.67–5.45	−0.21 to −0.20
*qSL-5A-3*	ZC	E11/E13/E14/B	190.20–191.00	6.80–11.15	6.25–9.96	0.33 to 0.43
*qSL-5A-4*	DS	E1/E3/E4	241.60–245.20	2.57–3.86	1.58–3.47	−0.17 to −0.15
*qSL-6A-1*	DS	E2/E3/E5/E6/B	165.80–177.60	4.37–9.06	2.27–4.17	0.17 to 0.21
*qSL-6B-4*	DS	E2/E3/E5/E6/B	180.80–198.60	8.93–35.46	4.00–21.30	−0.50 to −0.23
	GZ	E7/E9/E10/B	194.20–195.40	4.56–20.58	4.86–10.96	−0.43 to −0.23
	ZC	E13/E14	178.60–180.40	3.74–3.79	3.36–3.36	−0.26 to −0.25
*qSL-7A-2*	ZC	E13/E14	137.20–137.20	4.51–5.95	3.77–5.50	−0.30 to −0.27
*qSL-7A-3*	DS	E2/E5/B	152.00–152.20	4.15–17.49	1.62–8.52	0.13 to 0.32

*Pop, population; LOD, logarithm of odd; PVE, percentage of phenotypic variance explained; Add, additive effect; E1, 2012–2013 Beijing; E2, 2012–2013 Shijiazhuang; E3, 2013–2014 Beijing; E4, 2013–2014 Shijiazhuang; E5, 2014–2015 Beijing; E6, 2014–2015 Shijiazhuang; E7, 2012–2013 Anyang; E8, 2012–2013 Suixi; E9, 2013–2014 Anyang; E10, 2013–2014 Suixi; E11, Zhoukou2013; E12, Zhengzhou2013; E13, Zhoukou2014; E14, Zhengzhou2014; B, best linear unbiased estimation*.

In previous studies, QTL mapping using individual maps identified six, six, and nine stable QTLs in populations DS, GZ, and ZC, respectively (Gao et al., 2015; Li et al., 2018). This study detected all of them except *QSL.caas-5AL* in population ZC (Supplementary Table 7, Supplementary Figure 6). However, according to the linkage map constructed by Wen et al. (2017) for population ZC and the BLAST result, *QSL.caas-5AL* and *QSL.caas-5AL.1* tended to be the same. For the remaining 20 QTLs, 19 with stable effects were detected using the consensus map. *qSL-2D-1, qSL-2D-2*, and *qSL-5A-2* were detected only in one population using the individual maps, but all of them were detected in two populations using the consensus map (Table 4, Supplementary Table 7). With the consensus map, 10 other stable QTLs were identified for SL, i.e., *qSL-3A-4, qSL-3A-5, qSL-3B-5, qSL-4A-4, qSL-4B-1, qSL-4B-2, qSL-4D, qSL-5A-1, qSL-5A-4*, and *qSL-7A-3*, eight for population DS and two for population ZC.

### QTLs for TKW Detected From the Consensus Map and Comparison With Those From the Individual Maps

Using the consensus map, a total of 53 QTLs were detected for TKW (Supplementary Table 6), among which nine, three, and eight were stable in populations DS, GZ, and ZC, respectively (Figure 3, Table 5). *qTKW-4B-2* was repeatedly identified in populations DS and GZ with LOD scores ranging from 3.08 to 49.22, explaining 7.57–36.51% of the phenotypic variance. *qTKW-4B-2* had the largest LOD score, PVE and additive effect across environments in population DS. This QTL was co-localized with *qPH-4B-1*, corresponding to the dwarfing gene *Rht-B1*. All stable QTLs detected with the individual maps were also stable when detected with the consensus map (Supplementary Table 7, Supplementary Figure 7). There were other three stable TKW QTLs identified using the consensus map, i.e., *qTKW-1B-2, qTKW-2D-2*, and *qTKW-6B-3*. *qTKW-1B-2* was mapped on chromosome 1B at the interval of 588.36–591.14 Mb on the physical map, with LOD scores ranging from 4.70 to 45.92, and PVE values ranging from 2.37 to 22.43% in population DS. *qTKW-2D-2* was mapped on chromosome 2D at the interval of 523.15–555.13 Mb with LOD scores ranging from 3.91 to 14.69, and PVE values ranging from 4.94 to 12.32% in population ZC. *qTKW-6B-3* was mapped on chromosome 6B at the interval of 157.21–162.58 Mb with LOD scores varying from 3.66 to 4.91, and PVE values varying from 3.85 to 5.09% in population ZC.

**Table 5:** Stable QTLs identified for TKW in the three RIL populations, DS, GZ, and ZC using the consensus map.

**QTL**	**Pop**	**Environment**	**Position (cM)**	**LOD**	**PVE (%)**	**Add**
*qTKW-1B-2*	DS	E1/E2/E3/E6/B	44.40–45.00	4.70–45.92	2.37–22.43	0.93 to 3.72
*qTKW-2A-2*	DS	E2/E3/E4	125.00–125.00	2.57–7.27	1.32–5.90	0.70 to 1.15
*qTKW-2B-2*	DS	E4/E5/B	138.80–141.40	2.92–6.43	1.25–3.81	0.73 to 1.32
*qTKW-2D-2*	ZC	E11/E12/E13/E14/B	131.40–132.60	3.91–14.69	4.94–12.32	−1.64 to −1.00
*qTKW-3A-2*	GZ	E9/B	148.40–148.60	4.05–5.80	6.56–11.45	1.00 to 1.26
*qTKW-3D*	ZC	E11/E12/B	101.40–101.80	3.79–6.44	4.85–6.86	1.00 to 1.18
*qTKW-4A-1*	ZC	E11/E12/B	167.20–167.20	3.94–5.54	4.08–6.72	−1.17 to −0.91
*qTKW-4A-2*	DS	E1/E2/E6/B	200.00–213.40	2.51–4.57	0.89–3.47	−0.87 to −0.73
*qTKW-4B-2*	DS	E1/E2/E3/E4/E5/E6/B	75.00–76.00	13.28–49.22	8.81–36.51	1.60 to 3.78
	GZ	E7/E10/B	61.20–75.00	3.08–4.61	7.57–10.68	−1.43 to −1.09
*qTKW-5A-1*	ZC	E13/E14	65.40–84.60	3.92–4.67	3.02–3.53	−0.91 to −0.82
*qTKW-5A-3*	DS	E1/E2/E5/E6/B	116.40–124.40	3.83–7.46	2.53–5.05	1.03 to 1.27
*qTKW-5A-4*	GZ	E7/E8/E9/E10/B	106.20–112.00	2.69–4.68	6.36–8.64	−1.28 to −0.94
*qTKW-5D-1*	DS	E1/E2/E3/E4/E5/E6/B	49.20–54.80	6.23–11.44	2.38–7.58	1.12 to 1.66
*qTKW-6A-3*	ZC	E11/E12/E13/E14/B	98.60–102.20	5.67–16.80	7.37–14.28	−1.84 to −1.23
*qTKW-6B-3*	ZC	E11/E12/B	90.40–93.20	3.66–4.91	3.85–5.09	−1.01 to −0.82
*qTKW-6B-5*	DS	E1/E3/E5/E6/B	197.40–198.60	3.62–6.22	1.21–3.36	−1.17 to −0.86
*qTKW-7A-1*	ZC	E11/E12/E13/E14/B	140.40–141.20	2.72–4.77	2.04–5.71	−1.09 to −0.67
*qTKW-7B-3*	ZC	E13/E14	146.20–146.20	4.26–5.27	3.22–4.03	−0.98 to −0.85
*qTKW-7B-4*	DS	E1/E4/E5	168.80–171.40	5.79–18.41	3.10–12.11	1.06 to 2.36

*Pop, population; LOD, logarithm of odd; PVE, percentage of phenotypic variance explained; Add, additive effect; E1, 2012–2013 Beijing; E2, 2012–2013 Shijiazhuang; E3, 2013–2014 Beijing; E4, 2013–2014 Shijiazhuang; E5, 2014–2015 Beijing; E6, 2014–2015 Shijiazhuang; E7, 2012–2013 Anyang; E8, 2012–2013 Suixi; E9, 2013–2014 Anyang; E10, 2013–2014 Suixi; E11, Zhoukou2013; E12, Zhengzhou2013; E13, Zhoukou2014; E14, Zhengzhou2014; B, best linear unbiased estimation*.

### QTL Clusters for the Three Traits

As far as the stable QTLs across environments were concerned, 11 QTL clusters were identified and distributed on nine chromosomes (Supplementary Table 8), six of which affected two traits (i.e., *qClu-2D, qClu-4A-1, qClu-4A-2, qClu-4D, qClu-6A*, and *qClu-6B*), and five affected all the three traits (i.e., *qClu-3A-1, qClu-4B, qClu-5A-1, qClu-5A-2*, and *qClu-7A*). Eight clusters affected traits PH and SL. Among them, three clusters contained both PH and SL QTLs in population DS; one cluster contained both PH and SL QTLs in population ZC, and one cluster contained the closely linked PH and SL QTLs in populations DS and GZ. Each of the five clusters either increased or decreased both traits simultaneously. Genomic regions containing the stable QTLs for the three traits were located on chromosomes 3A, 4B, 5A, and 7A. The cluster on 4B was close to the Green Revolution gene *Rht-B1*. In cluster *qClu-5A-1*, QTLs affecting the three traits were consistently identified in populations DS and GZ, either increasing or decreasing the three traits simultaneously.

### Potential Applications of the Detected QTLs in Wheat Breeding

To explore the potential applications of the detected QTLs in wheat breeding, QTL genotypes and genotypic values of each RIL in the three populations were predicted on the three traits with stable QTLs identified using BLUE values across environments (Supplementary Tables 9–11). For convenience, for the two alleles at each QTL, one is called positive and the other one is called negative. Parental sources of the two alleles can be determined from the sign of the estimated additive effect of the QTL. Due to the varied objectives on different traits in breeding, it should be noted that the positive allele is not always favored and that the negative allele is not always un-favored. For PH, nine, eight, and eight stable QTLs were used for prediction in populations DS, GZ, and ZC, respectively. The 10 highest RILs possessed at least eight, seven, and seven positive alleles in the three populations, respectively, whereas the 10 lowest RILs had no more than two positive alleles (Supplementary Table 9). For SL, 14, 7, and 4 stable QTLs were used for prediction. The 10 highest RILs possessed at least nine, seven, and four positive alleles in the three populations, whereas the 10 lowest RILs had no more than four positive alleles in population DS, no positive allele in population GZ, and no more than 1 positive allele in population ZC (Supplementary Table 10). For TKW, seven, three, and six stable QTLs were used for prediction. The 10 highest RILs possessed at least six, three, and five positive alleles in the three populations, whereas the 10 lowest RILs had no more than 1 positive allele (Supplementary Table 11). RILs with the highest predicted genotypic values always had all the positive alleles for PH and TKW in the three mapping populations, and had all the positive alleles for SL in populations GZ and ZC. RILs with the lowest predicted genotypic values always had all the negative alleles for PH and SL in populations GZ and ZC, and had all the negative alleles for TKW in all the three mapping populations. For PH, all the 10 lowest RILs in population GZ and the 9 lowest RILs in population ZC contained the negative alleles at *qPH-4B-1* and *qPH-4D-1*, corresponding to genes *Rht-B1* and *Rht-D1*. For SL, *qSL-6B-4* was repeatedly identified in populations DS and GZ. Eighteen out of the 20 highest RILs in population DS and 36 highest RILs in population GZ possessed the positive allele at *qSL-6B-4*, while 17 out of the 20 lowest RILs in population DS and 38 lowest RILs in population GZ possessed the negative allele at *qSL-6B-4*. For TKW, *qTKW-4B-2* was consistently identified in populations DS and GZ. The 12.36% highest RILs in population DS and the 31.25% highest RILs in population GZ carried the positive allele at *qTKW-4B-2*, while the 17.45% lowest RILs in population DS and the 21.02% lowest RILs in population GZ carried the negative allele at *qTKW-4B-2*. Mean observed and predicted values of RILs having the positive allele at *qTKW-4B-2* were equal to 45.13 and 45.24 in population DS, and 47.11 and 47.95 in population GZ. In contrast, the observed means of RILs having the negative allele were equal to 42.68 and 40.4 in population DS, and 45.59 and 45.65 in population GZ.

Recombinant inbred lines with the predicted genotypic values on PH, SL, and TKW can serve for the choice of target genotypes meeting different breeding objectives, such as wheat cultivars with medium plant height, large spike length, and medium to high kernel weight. Given one target genotype, the predicted allelic combination of RILs can serve for the prediction of cross performance and the selection of suitable parental lines through simulation or other genomic prediction approaches (Yao et al., 2018).

### QTL Mapping in Simulated Populations

In 1,000 simulated populations, the estimated QTL positions and effects using the individual and consensus maps are shown in Table 6. With the increase in heritability, QTL detection powers were increased and the false discovery rate (FDR) was decreased in the three models using either the individual or consensus maps. Approximately unbiased estimation of QTL positions and effects was obtained for each defined model and heritability level. The confidence intervals of QTLs detected from the consensus map were much narrower, and the associated standard errors were much smaller than those from individual maps. Detection power was much lower for QTLs in linkage models II and III than that in the unlinked model I at the same heritability levels for both the individual and consensus maps. FDR was much higher in models II and III than in model I, indicating the complexity and difficulty in dissecting linked QTLs in genetic studies.

**Table 6:** Quantitative trait locus mapping results from 1,000 simulations using the individual and consensus maps in the three genetic models.

**Model**	***H*^**2**^^a^**	**Map**	**QTL**	**Pos. ± SE (cM)^b^**	**Add ± SE^c^**	**CIL ± SE^d^**	**LOD ± SE^e^**	**Power (%)**	**FDR (%)^f^**
I	0.05	Ind.	QTL1	34.32 ± 1.34	1.33 ± 0.22	3.55 ± 0.76	4.03 ± 1.41	31.5	44.44
		Cons.	QTL1	34.42 ± 1.36	1.32 ± 0.19	1.89 ± 0.35	3.97 ± 1.17	33.6	43.72
	0.1	Ind.	QTL1	34.42 ± 1.21	1.08 ± 0.21	3.30 ± 0.85	5.41 ± 2.10	71.1	25.16
		Cons.	QTL1	34.55 ± 1.27	1.09 ± 0.25	1.83 ± 0.41	5.62 ± 2.96	69.9	27.71
	0.2	Ind.	QTL1	34.40 ± 0.94	1.01 ± 0.16	2.85 ± 0.74	10.03 ± 3.02	89.3	12.45
		Cons.	QTL1	34.46 ± 1.12	1.01 ± 0.16	1.70 ± 0.41	10.07 ± 2.96	90.2	11.57
II	0.1	Ind.	QTL1	26.62 ± 1.37	2.03 ± 0.35	3.18 ± 1.05	5.61 ± 1.92	31.0	31.26
			QTL2	34.08 ± 1.32	1.99 ± 0.32	3.27 ± 0.97	5.27 ± 1.58	36.3	
		Cons.	QTL1	26.64 ± 1.29	2.07 ± 0.67	1.85 ± 0.37	6.00 ± 4.80	28.3	34.9
			QTL2	34.18 ± 1.37	1.98 ± 0.33	1.82 ± 0.42	5.33 ± 1.68	34.2	
	0.2	Ind.	QTL1	26.80 ± 1.21	1.88 ± 0.30	2.84 ± 0.83	9.93 ± 2.92	40.5	24.91
			QTL2	33.78 ± 1.11	1.84 ± 0.28	2.83 ± 1.02	9.54 ± 2.53	39.1	
		Cons.	QTL1	26.80 ± 1.21	1.87 ± 0.27	1.71 ± 0.42	9.96 ± 2.68	38.1	27.06
			QTL2	33.91 ± 1.20	1.83 ± 0.27	1.73 ± 0.46	9.52 ± 2.52	37.1	
	0.4	Ind.	QTL1	26.62 ± 1.09	1.33 ± 0.40	2.68 ± 0.84	13.06 ± 6.24	57.1	17.34
			QTL2	34.09 ± 1.03	1.39 ± 0.40	2.64 ± 0.86	13.99 ± 6.51	63.0	
		Cons.	QTL1	26.66 ± 1.130	1.36 ± 0.39	1.63 ± 0.40	13.67 ± 6.29	55.1	19.02
			QTL2	34.18 ± 1.180	1.39 ± 0.43	1.66 ± 0.39	14.19 ± 7.01	59.0	
III	0.1	Ind.	QTL1	26.11 ± 1.10	−1.04 ± 0.32	2.78 ± 0.95	9.51 ± 5.34	7.4	34.91
			QTL2	34.72 ± 1.11	1.04 ± 0.28	2.92 ± 0.87	9.26 ± 4.13	7.7	
		Cons.	QTL1	26.25 ± 1.04	−1.08 ± 0.35	1.68 ± 0.39	10.40 ± 6.04	7.3	40.93
			QTL2	34.82 ± 1.18	1.07 ± 0.31	1.71 ± 0.35	10.03 ± 4.85	8.0	
	0.2	Ind.	QTL1	26.19 ± 0.75	−0.98 ± 0.21	2.43 ± 0.63	16.07 ± 5.59	26.3	15.18
			QTL2	34.58 ± 0.77	0.99 ± 0.19	2.37 ± 0.67	16.24 ± 5.18	25.1	
		Cons.	QTL1	26.08 ± 0.75	−0.98 ± 0.2	1.54 ± 0.38	16.12 ± 5.36	27.3	16.05
			QTL2	34.58 ± 0.94	0.96 ± 0.18	1.51 ± 0.39	15.80 ± 5.17	27.1	
	0.4	Ind.	QTL1	26.33 ± 0.57	−0.95 ± 0.12	1.76 ± 0.49	30.92 ± 6.38	74.8	4.16
			QTL2	34.57 ± 0.59	0.94 ± 0.13	1.76 ± 0.51	30.80 ± 6.44	74.9	
		Cons.	QTL1	26.19 ± 0.66	−0.94 ± 0.13	1.25 ± 0.36	30.67 ± 6.83	77.2	5.23
			QTL2	34.51 ± 0.82	0.94 ± 0.13	1.27 ± 0.35	30.65 ± 6.51	76.9	

a *Heritability in broad sense*.

b *Position in cM and the associated standard error*.

c *Additive effect and the associated standard error*.

d *Confidence interval length and the associated standard error*.

e *LOD scores and the associated standard error*.

f *False discovery rate*.

*Ind., individual map; Cons., consensus map*.

## Discussion

### Computer Tools in Consensus Map Construction

Two strategies have been adopted for consensus map construction in previous studies (Endelman and Plomion, 2014). The first one is based on the raw data of multiple mapping populations, and has been implemented in software MultiPoint (Ronin et al., 2012) and JoinMap (Van Ooijen, 2006). The second one is based on individual linkage maps previously constructed, and has been implemented in software BioMercator (Arcade et al., 2004), MergeMap (Wu et al., 2010), LPmerge (Endelman and Plomion, 2014), and QTL IciMapping (Meng et al., 2015). The first strategy is usually time-consuming when dealing with a large number of markers (Wu et al., 2010), which has drastically restricted the use of a large number of markers in the consensus map. The second strategy highly depends on the quality of individual maps and sometimes may result in maps with unreasonable length (Cavanagh et al., 2013; Wang et al., 2014; Wingen et al., 2017).

With the development of high-throughput sequencing technology, markers that can be used in genotyping mapping populations are growing rapidly. A large amount of markers brings a great challenge to consensus map construction, especially when raw genotypic data are used. The two raw data-based software packages mentioned above cannot deal with such a large number of markers used in this study. For example, both packages cannot generate a consensus map for chromosome 5B, which harbored 929, 1,406, and 1,508 SNPs in populations DS, GZ, and ZC, respectively. Map-based method only utilizes marker distances between adjacent markers, which may result in an inaccurate estimation of recombination frequency between markers especially when the order of markers changes on the consensus map. The CLA algorithm is a raw data-based method used in this study to deal with a large amount of markers. The combined recombination frequency between any pair of markers was calculated from the estimates in individual mapping populations. The estimated recombination frequencies are recorded in computer memory. Therefore, time can be greatly saved in computing.

### Quality of the Consensus Map

The great number of markers and bins contained in the consensus map provided higher saturation of markers and better genome coverage, and expanded the length of the map. Previous studies have shown that increased recombination events and map resolution with an increased number of markers and density could contribute to longer map length (Ferreira et al., 2006; Wingen et al., 2017). The longer map length may also suffer from chromosomal structure differences in different mapping populations and the ordering algorithm used. Compared with the A and B genomes, the D genome had fewer unique markers, larger gaps, and shorter map length, which have been previously reported in both consensus and individual maps in wheat (Wang et al., 2014; Li et al., 2015; Guan et al., 2018).

Collinearity was high between the genetic and physical positions. Marker order on the consensus and physical maps was highly correlated at the genome-wide level, but lower collinearity was sometimes observed in some chromosomal regions, which was also reported previously (Wingen et al., 2017). Of the 19,320 SNPs on the consensus map that had physical positions, on average there were 55.17% SNPs arranged in the same order as those on the corresponding chromosomes of the physical maps, ranging from 36.3 on chromosome 6A to 75.68% on chromosome 4D (Table 2). A higher proportion of the completely consistent marker order was found in the D genome (63.95%) than those in the A genome (52.32%) and the B genome (49.22%), which may be explained by the lower recombination on the D genome. The lower recombination events on the D genome contributed to lower sequence variability and had a weaker influence on the decay of syntenic block size. Some chromosomal structural variations were observed on the consensus map, such as intra-chromosomal translocation and inversion. For example, inversion happened around 22–25 Mb on chromosome 1A, and translocation occurred between regions around 88–93 and 106–109 Mb on chromosome 2A. The collinearity between marker orders in genetic and physical maps is often disturbed by the macrostructural variations in wheat, especially for consensus maps that are constructed from multiple populations. Local disorder of markers could also be caused by the variation of gene order in parents and genotyping errors.

The distribution of meiotic recombination events showed that recombination happened much more frequently in the distal chromosomal regions, and that recombination tended to be suppressed near the centromeres, which was consistent with previous studies [Sourdille et al., 2004; International Wheat Genome Sequencing Consortium (IWGSC), 2018]. Collinearity analysis also showed that some markers might have conservative orders across populations, since their relative orders were consistent on the physical and genetic maps. Comparative analysis among the consensus, physical, and individual maps indicated the reliability of the consensus map constructed with the CLA algorithm.

### Comparison of the Detected QTLs With Studies on Other Mapping Populations

In this study, eight stable PH QTLs were detected with the consensus map but not with the individual maps (Table 7). Guan et al. (2018) reported a PH QTL on chromosome 4D at the physical interval of 37.05–62.94 Mb, and Ren et al. (2021) reported a PH QTL on the same chromosome at the physical interval of 47.44–67.64Mb. *qPH-4D-2* (chr4D:32.97–65.01 Mb) was overlapped with the loci reported by Guan et al. (2018) and Ren et al. (2021). *qPH-6A-1* was located within the physical region as reported by Zanke et al. (2014). *qPH-6A-2* was mapped on chromosome 6A at the interval of 610.97–613.55 Mb. Similarly, Pang et al. (2020) detected a PH QTL on chromosome 6A at the interval of 609.3–609.9 Mb (IWGSC RefSeq v1.0). *qPH-6D-2* was located at the same marker interval of a PH QTL that was first reported and validated to be stable in two wheat populations by Wang et al. (2020). To the best knowledge of the authors, stable QTLs *qPH-2D-1, qPH-2D-3, qPH-3B-2*, and *qPH-7A* identified in this study were likely to be novel for PH. The increased marker density in the consensus map contributed to the detection of these novel QTLs.

**Table 7:** Quantitative trait loci for PH, SL, and TKW detected with the consensus map but not by the individual maps in the three RIL populations, DS, GZ, and ZC.

**Trait**	**QTL**	**Pop**	**Environment**	**Physical interval (Mb)*^a^***	**Neighboring loci in previous studies**
PH	*qPH-2D-1*	ZC	E13/E14/B	344.29–426.06	*TaERF8-2D*, Zhang et al., 2020b
	*qPH-2D-3*	DS	E1/E2/E3/E4/B	617.78–631.92	
	*qPH-3B-2*	GZ	E7/E10/B	842.16–844.72	
	*qPH-4D-2*	GZ	E7/E8/E9/E10/B	32.97–65.01	*QPh.cau-4D.2*, Guan et al., 2018
		ZC	E11/E12/E13/E14/B		*QPh.sau-4D*, Ren et al., 2021
	*qPH-6A-1*	GZ	E7/E9/B	600.13–600.63	Zanke et al., 2014
	*qPH-6A-2*	DS	E1/E3/B	610.97–613.55	*qPH6A.4*, Pang et al., 2020
	*qPH-6D-2*	ZC	E11/E13/E14/B	337.17–361.16	*QPh.sicau-6D*, Wang et al., 2020
	*qPH-7A*	DS	E1/E2/E3/E5/B	611.92–621.35	
SL	*qSL-3A-4*	DS	E2/E5/B	656.58–663.11	
	*qSL-3A-5*	ZC	E12/E14/B	722.85–748.34	
	*qSL-3B-5*	DS	E2/E4/E6/B	761.90–774.47	*QSL-3B.2*, Hu et al., 2020
	*qSL-4A-4*	DS	E1/E2/E5/B	719.47–750.82	*qSL4A.3*, Pang et al., 2020
	*qSL-4B-1*	DS	E1/E2/B	6.94–10.81	
	*qSL-4B-2*	DS	E1/E2/E3/E4/E5/E6/B	34.98–49.80	*QSl.sdau-4B*, Deng et al., 2011*qSL4B.1*, Pang et al., 2020
	*qSL-4D*	DS	E1/E2/E5/B	65.53–121.40	
	*qSL-5A-1*	ZC	E13/E14	437.35–445.46	
	*qSL-5A-4*	DS	E1/E3/E4	671.95–681.28	*qSL5A.2*, Pang et al., 2020
	*qSL-7A-3*	DS	E2/E5/B	647.11–648.26	
TKW	*qTKW-1B-2*	DS	E1/E2/E3/E6/B	588.36–591.14	*BS00039740_51*, Gerard et al., 2019
	*qTKW-2D-2*	ZC	E11/E12/E13/E14/B	523.15–555.13	*AX-109775854*, Zhang et al., 2020c
	*qTKW-6B-3*	ZC	E11/E12/B	157.21–162.58	*IWB61228-6B*, Cook et al., 2021

a *Physical positions for the flanking markers of QTLs based on IWGSC_RefSeq v2.0*.

*E1, 2012–2013 Beijing; E2, 2012–2013 Shijiazhuang; E3, 2013–2014 Beijing; E4, 2013–2014 Shijiazhuang; E5, 2014–2015 Beijing; E6, 2014–2015 Shijiazhuang; E7, 2012–2013 Anyang; E8, 2012–2013 Suixi; E9, 2013–2014 Anyang; E10, 2013–2014 Suixi; E11, Zhoukou2013; E12, Zhengzhou 2013; E13, Zhoukou 2014; E14, Zhengzhou 2014; B, best linear unbiased estimation*.

For spike length, 10 QTLs were detected with the consensus map but not with the individual maps (Table 7). Among them, a stable QTL in population DS, i.e., *qSL-4B-2* explaining 3.60–16.99% of the phenotypic variance, was close to the Green Revolution gene *Rht-B1*. A number of previous studies have revealed that *Rht-B1* has a pleiotropic effect on PH, SL, and TKW (Schulthess et al., 2017; Sun et al., 2017; Li et al., 2018). QTL cluster *qClu-4B* in which *qSL-4B-2* was located affected all three traits (Supplementary Table 8). However, no stable PH QTL in *qClu-4B* was detected in population DS, indicating that *qSL-4B-2* may not be the same as *Rht-B1*. One SL QTL, i.e., *QSl.sdau-4B*, different from but close to *Rht-B1*, was precisely mapped and verified by Deng et al. (2011), which did not affect PH either. *SL-4B-2* was located in a similar position as *QSl.sdau-4B*, and was also in a similar physical position of *qSL4B.1* (chr4B: 36.7–37.8 Mb) reported by Pang et al. (2020). For the remaining nine QTLs, *qSL-3B-5* was mapped on chromosome 3B at the interval of 761.9–774.47 Mb, which was in the similar physical interval (chr3B: 771.94–788.06 Mb) as reported by Hu et al. (2020); *qSL-4A-4* and *qSL-5A-4* were close to those reported in Pang et al. (2020). Six SL QTLs were likely to be novel because of increased power when using the consensus map in QTL mapping, i.e., *qSL-3A-4, qSL-3A-5, qSL-4B-1, qSL-4D, qSL-5A-1*, and *qSL-7A-3*.

Compared with the individual maps, three other TKW QTLs were stably identified using the consensus map (Table 7), i.e., *qTKW-1B-2, qTKW-2D-2*, and *qTKW-6B-3*, which were in similar positions as those reported by Gerard et al. (2019), Zhang et al. (2020c), and Cook et al. (2021), respectively.

For the three traits, a total of 21 QTLs were identified using the consensus map but not the individual maps. Among them, 11 QTLs are consistent with those from previous studies on other mapping populations, and 10 QTLs are likely to be novel. Most of the 11 QTLs were first reported in recent years using high-density linkage maps, indicating that the increase in marker density improved the power of QTL detection. For the novel QTLs, six of them that control PH or SL were included in the cluster that harbored closely linked PH and SL QTLs (Supplementary Table 8). The PH of the wheat plant is equal to SL plus the lengths of all internodes above the ground. Theoretically, loci associated with SL may affect PH as well, which has been validated by some studies (Buerstmayr et al., 2011; Lv et al., 2014; Xu et al., 2014; Jahani et al., 2019; Chen et al., 2020). Furthermore, four novel SL QTLs were close to PH QTLs that have been reported using individual maps or other independent studies, indicating the reliability of the novel QTLs on SL or PH. Gene *TaERF8* was identified to be associated with PH and yield in wheat, and has been cloned from the wheat cultivar Chinese Spring (Zhang et al., 2020b), one parental line of population ZC. *TaERF8-2D* (chr2D: 368.21 Mb) was located in the flanking marker interval of *qPH-2D-1*, which was stably detected in population ZC in the three tested environments and in population DS in two tested environments. *TaERF8-2D* may be a candidate gene for *qPH-2D-1*. Annotations of gene functions were also performed for these novel QTLs based on the wheat reference sequence annotation database (IWGSC Annotation v1.1) as listed in Supplementary Table 12. The annotation information will facilitate the future fine mapping, map-based cloning, and functional analysis of the novel QTLs identified in this study.

### Relationship Between QTLs for Phenotypically Correlated Traits PH and SL

Plant height is an important agronomic trait highly related to lodging resistance and harvest index in wheat. SL is highly related to grain yield by affecting kernel number and spike morphology (Donmez et al., 2001). Plants with suitable PH and larger spike are desirable in wheat breeding. Nine of the 21 stable PH QTLs were close to the stable SL QTLs (Supplementary Table 8), contributing to the genetic correlation between the two traits. PH and SL were positively correlated by phenotypic analysis in population DS, but the correlation was non-significant in the other two populations. In this study, closely linked PH and SL QTLs identified in the same population always had genetic effects at the same directions on both traits. Similar instances have been reported in previous studies (Buerstmayr et al., 2011; Lv et al., 2014; Xu et al., 2014; Jahani et al., 2019; Chen et al., 2020). Considering that some QTLs for SL may also affect PH, we speculated that the closely linked PH and SL QTLs are more likely to be the same genetic loci and have the same effect directions. However, whether the closely linked QTLs on PH and SL belong to the same chromosomal loci with pleiotropic effects or different closely-linked loci needs further investigation and is beyond the scope of this study.

### Further Analysis for a Major PH QTL Located on Chromosome 4DS

For plant height, only one QTL was detected on chromosome 4DS using the individual maps in populations GZ and ZC, but two stable QTLs, i.e., *qPH-4D-1* and *qPH-4D-2*, were identified using the consensus map in the same two populations, which were linked in the coupling phase (Table 3, Supplementary Table 7). The BLAST results indicated that *qPH-4D-1* was co-localized with the dwarfing gene *Rht-D1*. *qPH-4D-2* explained 8.39–10.9 and 5.03–12.03% of the phenotypic variance across environments in populations GZ and ZC, respectively. The alleles decreasing PH were from parents Zhoumai16 in population GZ and Zhou 8425B in population ZC.

Guan et al. (2018) reported two QTLs that were also linked in the coupling phase and located in similar positions as *qPH-4D-1* and *qPH-4D-2*. *qPH-4D-2* was detected in four environments and with BLUE values across eight environments in Guan et al. (2018). In addition, *qPH-4D-2* was closely linked with marker wsnp_Ex_c683_1341113, which was also observed in Guan et al. (2018). As reported by Ren et al. (2021), *qPH-4D-2* was detected in the similar position between SNPs AX-89692818 and AX-109606880 across environments. Therefore, it is highly possible that *qPH-4D-2* is a novel semi-dwarfing gene. The common marker wsnp_Ex_c683_1341113 was located at about 54.4 Mb on chromosome 4D (IWGSC RefSeq v1.0; IWGSC, 2018). A high confidence putative gene, *TraesCS4D02G076400* (50,888,586–50,889,461 bp), is located around the marker and in the confidence interval of *qPH-4D-2*, with the annotation of encoding gibberellin regulated protein (IWGSC RefSeq v1.1 annotation; IWGSC, 2018). Gibberellin is an essential endogenous regulator in plant growth. The well-known dwarfing genes *Rht-B1b* and *Rht-D1b* regulate DELLA proteins in gibberellin signaling to reduce the response to gibberellin (Peng et al., 1999). The gibberellin-sensitive gene *Rht8* was also widely used in regulating PH in wheat (Gasperini et al., 2012). Gene *TraesCS4D02G076400* in wheat was annotated to gene *GAST1* (UniProtKB/TrEMBL; Acc:C8C4P9), first reported in tomato to encode the gibberellins-stimulated transcript (Shi et al., 1992). *GAST1* belongs to the gibberellic acid-stimulated *Arabidopsis* (*GASA*) family, which plays important roles in plant growth and development, such as stem growth, plant height, and grain length, width, and weight (de la Fuente et al., 2006; Nahirñak et al., 2012a,b; Shi et al., 2020). Furthermore, *qPH-4D-2* was detected in two populations in this study, one from the cross between Zhou 8425B and Chinese Spring. *TraesCS4D02G076400* had high RNA expression levels in Chinese Spring in different tissues and development stages (expVIP, http://www.wheat-expression.com/). Therefore, *TraesCS4D02G076400* is likely to be the candidate gene for *qPH-4D-2*. PH is a crucial trait for morphogenesis and grain yield in wheat. The newly discovered PH QTL on chromosome 4DS in this study may enrich the genetic resources in breeding for semi-dwarfing wheat. Reasons that *qPH-4D-2* was not identified by the individual could be the short distance between the QTL and *Rht-D1*, and the lower marker density around the two QTLs in individual mapping populations.

### Advantages of Using Consensus Map in QTL Mapping

Due to the limited number of crossing-overs and limited genetic variation in individual populations, linkage maps constructed from individual mapping populations usually have a large number of co-localized markers and low marker density. A consensus map combines the genetic information included in multiple populations and provides a better genomic coverage with higher marker density (Maccaferri et al., 2015; Allen et al., 2017). A consensus map of higher density offers the chance to map QTLs to narrower chromosomal intervals, which will facilitate the discovery of causal genes and the identification of closely linked markers for MAS. Simulation results conducted in this study confirmed that the use of a consensus map with higher marker density reduced the confidence interval of detected QTLs.

Even for the same trait, QTLs detected in different populations using their own genetic maps sometimes are hardly compared and synthesized, because of the unshared markers and variations in the genetic background (Sukumaran et al., 2015). Comparisons on QTL positions estimated from different populations are usually conducted by anchoring the linked markers to the genome assembly. However, genome sequences usually have wide variations between parental varieties, and the anchor information to the genome sequence may not be completely accurate. A consensus map provides the direct comparison for QTLs detected from different populations, which is important, particularly in species lacking a completely sequenced reference genome. In this study, we demonstrated that QTL mapping using a consensus map can better identify common and stable QTLs across populations and environments. For example, *Rht-B1* and *Rht-D1*, which had been cloned, were the two genes reducing plant height in wheat (Peng et al., 1999). Each of them was located almost in the same position in two populations on the consensus map. *qPH-5A-2, qSL-2D-2, qSL-5A-2*, and *qTKW-4B-2* were detected in populations DS and GZ; *qPH-2B-2* was detected in populations DS and ZC; *qPH-4B-1, qPH-4D-1, qPH-4D-2*, and *qSL-2D-1* were identified in populations GZ and ZC; *qSL-6B-4* was detected in all the three populations. The common QTLs identified in multiple populations reflected the stable genetic effects of QTLs in different genetic backgrounds, which might be more valuable in breeding.

The genetic relationship among PH and SL QTLs as observed in this study, showed that QTL mapping using the consensus map can also facilitate the comparison across the correlated traits, and therefore provide the opportunity to understand the genetic correlation between phenotypically correlated traits and identify the QTL-rich genomic regions. Moreover, the consensus map also provides the chance to detect common QTLs with smaller effects occurring in different populations.

Further studies may still be needed to determine the key factors affecting the accuracy of consensus map construction and subsequent QTL mapping, such as proportion of common markers shared by multiple mapping populations, inconsistency degree of marker orders in individual populations, population-specific recombination frequencies, and the optimum algorithm used to construct the consensus map. In addition to bi-parental populations, as have been used in this study, multi-parental populations have been developed in recent years in crops together with suitable genetic analysis methods (Gardner et al., 2016; Zhang et al., 2017, 2019; Shi et al., 2019; Qu et al., 2020). In theory, a consensus map can also be constructed by combining a number of bi-parental and multi-parental populations, when common markers are shared by these populations.

In conclusion, the consensus map constructed for this study allows for systematic QTL mapping studies, and comparison and clustering of mapping results in wheat genetic studies. The QTL mapping based on the consensus map resulted in higher accuracy, narrower confidence interval, and a larger QTL number. The stable QTLs across tested environments and mapping populations, and the predicted QTL genotypes and genotypic values can be used to select wheat cultivars with suitable PH, large SL, and medium to high kernel weight. SNPs closely linked with these stable QTLs can be used to select suitable genetic materials and make suitable crosses in wheat breeding programs. SNPs closely linked to traits can also be converted into Kompetitive allele-specific PCR (KASP) markers (Kaur et al., 2021) and then used for large-scale genotyping to screen desirable individuals in segregating breeding populations.

## Data Availability Statement

The original contributions presented in the study are included in the article/Supplementary Material, further inquiries can be directed to the corresponding author/s.

## Author Contributions

LZ and JW conceived and designed the research. PQ and LZ conducted data analysis. FG, WW, JL, and XX developed the populations, performed SNP genotyping, and conducted field trials. PQ, JW, and LZ wrote, drafted, and revised the manuscript. XX and HP provided guidance on data analysis and revised the manuscript. All the authors read and approved the final version of the manuscript for publication.

## Conflict of Interest

The authors declare that the research was conducted in the absence of any commercial or financial relationships that could be construed as a potential conflict of interest.

## Publisher's Note

All claims expressed in this article are solely those of the authors and do not necessarily represent those of their affiliated organizations, or those of the publisher, the editors and the reviewers. Any product that may be evaluated in this article, or claim that may be made by its manufacturer, is not guaranteed or endorsed by the publisher.

## References

[B1] Allen A. M. Winfield M. O. Burridge A. J. Downie R. C. Benbow H. R. Barker G. L.. (2017). Characterization of a Wheat Breeders' Array suitable for high-throughput SNP genotyping of global accessions of hexaploid bread wheat (*Triticum aestivum*). Plant Biotechnol. J. 15, 390–401. 10.1111/pbi.12635 27627182 PMC5316916

[B2] Arcade A. Labourdette A. Falque M. Mangin B. Chardon F. Charcosset A.. (2004). BioMercator: integrating genetic maps and QTL towards discovery of candidate genes. Bioinformatics 20, 2324–2326. 10.1093/bioinformatics/bth230 15059820

[B3] Buerstmayr M. Lemmens M. Steiner B. Buerstmayr H. (2011). Advanced backcross QTL mapping of resistance to Fusarium head blight and plant morphological traits in a *Triticum macha*× *T. aestivum* population. Theor. Appl. Genet. 123:293. 10.1007/s00122-011-1584-x 21479934 PMC3114081

[B4] Cavanagh C. R. Chao S. Wang S. Huang B. E. Stephen S. Kiani S.. (2013). Genome-wide comparative diversity uncovers multiple targets of selection for improvement in hexaploid wheat landraces and cultivars. Proc. Natl. Acad. Sci. U.S.A. 110, 8057–8062. 10.1073/pnas.1217133110 23630259 PMC3657823

[B5] Chen H. Boutros P. C. (2011). VennDiagram: a package for the generation of highly-customizable Venn and Euler diagrams in R. BMC Plant Biol. 12:35. 10.1186/1471-2105-12-35 21269502 PMC3041657

[B6] Chen W. Sun D. Li R. Wang S. Shi Y. Zhang W.. (2020). Mining the stable quantitative trait loci for agronomic traits in wheat (*Triticum aestivum* L.) based on an introgression line population. BMC Plant Biol. 20:275. 10.1186/s12870-020-02488-z 32539793 PMC7296640

[B7] Cook J. Acharya R. Martin J. Blake N. Khan I. Heo H. Y.. (2021). Genetic analysis of stay-green, yield, and agronomic traits in spring wheat. Crop Sci. 61, 383–395. 10.1002/csc2.20302

[B8] de la Fuente J. I. Amaya I. Castillejo C. Sánchez-Sevilla J. F. Quesada M. A. Botella M. A.. (2006). The strawberry gene *FaGAST* affects plant growth through inhibition of cell elongation. J. Exp. Bot. 57, 2401–2411. 10.1093/jxb/erj213 16804055

[B9] Deng S. Wu X. Wu Y. Zhou R. Wang H. Jia J.. (2011). Characterization and precise mapping of a QTL increasing spike number with pleiotropic effects in wheat. Theor. Appl. Genet. 122, 281–289. 10.1007/s00122-010-1443-1 20872211

[B10] Donmez E. Sears R. Shroyer J. Paulsen G. (2001). Genetic gain in yield attributes of winter wheat in the Great Plains. Crop Sci. 41, 1412–1419. 10.2135/cropsci2001.4151412x

[B11] Endelman J. B. Plomion C. (2014). LPmerge: an R package for merging genetic maps by linear programming. Bioinformatics 30, 1623–1624. 10.1093/bioinformatics/btu091 24532720

[B12] Ferreira A. da Silva M. F. Cruz C. D. (2006). Estimating the effects of population size and type on the accuracy of genetic maps. Genet. Mol. Biol. 29, 187–192. 10.1590/S1415-47572006000100033

[B13] Gao F. Wen W. Liu J. Rasheed A. Yin G. Xia X.. (2015). Genome-wide linkage mapping of QTL for yield components, plant height and yield-related physiological traits in the Chinese wheat cross Zhou 8425B/Chinese Spring. Front. Plant Sci. 6:1099. 10.3389/fpls.2015.01099 26734019 PMC4683206

[B14] Gardner K. A. Wittern L. M. Mackay I. J. (2016). A highly recombined, high-density, eight-founder wheat MAGIC map reveals extensive segregation distortion and genomic locations of introgression segments. Plant Biotechnol. J. 14, 1406–1417. 10.1111/pbi.12504 26801965 PMC4985697

[B15] Gasperini D. Greenland A. Hedden P. Dreos R. Harwood W. Griffiths S. (2012). Genetic and physiological analysis of *Rht8* in bread wheat: an alternative source of semi-dwarfism with a reduced sensitivity to brassinosteroids. J. Exp. Bot. 63:4419. 10.1093/jxb/ers292 22791821 PMC3421992

[B16] Gerard G. S. Alqudah A. Lohwasser U. Börner A. Simón M. R. (2019). Uncovering the genetic architecture of fruiting efficiency in bread wheat: a viable alternative to increase yield potential. Crop Sci. 59, 1853–1869. 10.2135/cropsci2018.10.0639

[B17] Guan P. Lu L. Jia L. Kabir M. R. Zhang J. Lan T.. (2018). Global QTL analysis identifies genomic regions on chromosomes 4A and 4B harboring stable loci for yield-related traits across different environments in wheat (*Triticum aestivum* L.). Front. Plant Sci. 9:529. 10.3389/fpls.2018.00529 29922302 PMC5996883

[B18] Hu J. Wang X. Zhang G. Jiang P. Chen W. Hao Y.. (2020). QTL mapping for yield-related traits in wheat based on four RIL populations. Theor. Appl. Genet. 133, 917–933. 10.1007/s00122-019-03515-w 31897512

[B19] International Wheat Genome Sequencing Consortium (IWGSC) (2018). Shifting the limits in wheat research and breeding using a fully annotated reference genome. Science 361:eaar7191. 10.1126/science.aar7191 30115783

[B20] Jahani M. Mohammadi-Nejad G. Nakhoda B. Rieseberg L. H. (2019). Genetic dissection of epistatic and QTL by environment interaction effects in three bread wheat genetic backgrounds for yield-related traits under saline conditions. Euphytica 215:103. 10.1007/s10681-019-2426-1

[B21] Kaur J. Kaur J. Dhillon G. S. Kaur H. Singh J. Bala R.. (2021). Characterization and mapping of spot blotch in *Triticum durum–Aegilops speltoides* introgression lines using SNP markers. Front. Plant Sci. 12:650400. 10.3389/fpls.2021.650400 34122476 PMC8193842

[B22] Li F. Wen W. He Z. Liu J. Jin H. Cao S.. (2018). Genome-wide linkage mapping of yield-related traits in three Chinese bread wheat populations using high-density SNP markers. Theor. Appl. Genet. 131, 1903–1924. 10.1007/s00122-018-3122-6 29858949

[B23] Li H. Vikram P. Singh R. P. Kilian A. Carling J. Song J.. (2015). A high density GBS map of bread wheat and its application for dissecting complex disease resistance traits. BMC Genomics 16:216. 10.1186/s12864-015-1424-5 25887001 PMC4381402

[B24] Li H. Zhang L. Wang J. (2012). Estimation of statistical power and false discovery rate of QTL mapping methods through computer simulation. Chin. Sci. Bull. 57, 2701–2710. 10.1007/s11434-012-5239-3

[B25] Liu Y. Salsman E. Wang R. Galagedara N. Zhang Q. Fiedler J. D.. (2020). Meta-QTL analysis of tan spot resistance in wheat. Theor. Appl. Genet. 133, 2363–2375. 10.1007/s00122-020-03604-1 32436020

[B26] Liu Y. Zeng Z.-B. (2000). A general mixture model approach for mapping quantitative trait loci from diverse cross designs involving multiple inbred lines. Genet. Res. 75, 345–355. 10.1017/S0016672300004493 10893870

[B27] Lorieux M. (2012). MapDisto: fast and efficient computation of genetic linkage maps. Mol. Breed. 30, 1231–1235. 10.1007/s11032-012-9706-y

[B28] Lv C. Song Y. Gao L. Yao Q. Zhou R. Xu R.. (2014). Integration of QTL detection and marker assisted selection for improving resistance to Fusarium head blight and important agronomic traits in wheat. Crop J. 2, 70–78. 10.1016/j.cj.2013.10.004

[B29] Maccaferri M. Ricci A. Salvi S. Milner S. G. Noli E. Martelli P. L.. (2015). A high-density, SNP-based consensus map of tetraploid wheat as a bridge to integrate durum and bread wheat genomics and breeding. Plant Biotechnol. J. 13, 648–663. 10.1111/pbi.12288 25424506

[B30] Meng L. Li H. Zhang L. Wang J. (2015). QTL IciMapping: integrated software for genetic linkage map construction and quantitative trait locus mapping in biparental populations. Crop J. 3, 269–283. 10.1016/j.cj.2015.01.001

[B31] Nahirñak V. Almasia N. I. Fernandez P. V. Hopp H. E. Estevez J. M. Carrari F.. (2012a). Potato snakin-1 gene silencing affects cell division, primary metabolism, and cell wall composition. Plant Physiol. 158, 252–263. 10.1104/pp.111.186544 22080603 PMC3252113

[B32] Nahirñak V. Almasia N. I. Hopp H. E. Vazquez-Rovere C. (2012b). Snakin/GASA proteins: involvement in hormone crosstalk and redox homeostasis. Plant Signal. Behav. 7, 1004–1008. 10.4161/psb.20813 22836500 PMC3474668

[B33] Ouellette L. A. Reid R. W. Blanchard S. G. Brouwer C. R. (2018). LinkageMapView—rendering high-resolution linkage and QTL maps. Bioinformatics 34, 306–307. 10.1093/bioinformatics/btx576 28968706 PMC5860205

[B34] Pang Y. Liu C. Wang D. Amand P. S. Bernardo A. Li W.. (2020). High-resolution genome-wide association study identifies genomic regions and candidate genes for important agronomic traits in wheat. Mol. Plant 13, 1311–1327. 10.1016/j.molp.2020.07.008 32702458

[B35] Peng J. Richards D. E. Hartley N. M. Murphy G. P. Devos K. M. Flintham J. E.. (1999). ‘Green revolution’genes encode mutant gibberellin response modulators. Nature 400, 256–261. 10.1038/22307 10421366

[B36] Qu P. Shi J. Chen T. Chen K. Shen C. Wang J.. (2020). Construction and integration of genetic linkage maps from three multi-parent advanced generation inter-cross populations in rice. Rice 13:13. 10.1186/s12284-020-0373-z 32060661 PMC7021868

[B37] Rasheed A. Wen W. Gao F. Zhai S. Jin H. Liu J.. (2016). Development and validation of KASP assays for genes underpinning key economic traits in bread wheat. Theor. Appl. Genet. 129, 1843–1860. 10.1007/s00122-016-2743-x 27306516

[B38] Ren T. Fan T. Chen S. Li C. Chen Y. Ou X.. (2021). Utilization of a Wheat55K SNP array-derived high-density genetic map for high-resolution mapping of quantitative trait loci for important kernel-related traits in common wheat. Theor. Appl. Genet. 134, 807–821. 10.1007/s00122-020-03732-8 33388883

[B39] Ronin Y. Mester D. Minkov D. Belotserkovski R. Jackson B. Schnable P.. (2012). Two-phase analysis in consensus genetic mapping. G3-Genes Genomes Genet. 2, 537–549. 10.1534/g3.112.002428 22670224 PMC3362937

[B40] Schulthess A. W. Reif J. C. Ling J. Plieske J. Kollers S. Ebmeyer E.. (2017). The roles of pleiotropy and close linkage as revealed by association mapping of yield and correlated traits of wheat (*Triticum aestivum* L.). J. Exp. Bot. 68, 4089–4101. 10.1093/jxb/erx214 28922760 PMC5853857

[B41] Sharp P. Kreis M. Shewry P. Gale M. (1988). Location of β-amylase sequences in wheat and its relatives. Theor. Appl. Genet. 75, 286–290. 10.1007/BF00303966

[B42] Shi C. L. Dong N. Q. Guo T. Ye W. W. Shan J. X. Lin H. X. (2020). A quantitative trait locus *GW6* controls rice grain size and yield through the gibberellin pathway. Plant J. 103, 1174–1188. 10.1111/tpj.14793 32365409

[B43] Shi J. Wang J. Zhang L. (2019). Genetic mapping with background control for quantitative trait locus (QTL) in 8-parental pure-line populations. J. Hered. 110, 880–891. 10.1093/jhered/esz050 31419284 PMC6916664

[B44] Shi L. Gast R. T. Gopalraj M. Olszewski N. E. (1992). Characterization of a shoot-specific, GA3-and ABA-regulated gene from tomato. Plant J. 2, 153–159. 10.1046/j.1365-313X.1992.t01-39-00999.x 1302047

[B45] Somers D. J. Isaac P. Edwards K. (2004). A high-density microsatellite consensus map for bread wheat (*Triticum aestivum* L.). Theor. Appl. Genet. 109, 1105–1114. 10.1007/s00122-004-1740-7 15490101

[B46] Sourdille P. Singh S. Cadalen T. Brown-Guedira G. L. Gay G. Qi L.. (2004). Microsatellite-based deletion bin system for the establishment of genetic-physical map relationships in wheat (*Triticum aestivum* L.). Funct. Integr. Genomics 4, 12–25. 10.1007/s10142-004-0106-1 15004738

[B47] Stam P. (1993). Construction of integrated genetic linkage maps by means of a new computer package: join map. Plant J. 3, 739–744. 10.1111/j.1365-313X.1993.00739.x

[B48] Sukumaran S. Dreisigacker S. Lopes M. Chavez P. Reynolds M. P. (2015). Genome-wide association study for grain yield and related traits in an elite spring wheat population grown in temperate irrigated environments. Theor. Appl. Genet. 128, 353–363. 10.1007/s00122-014-2435-3 25490985

[B49] Sun C. Zhang F. Yan X. Zhang X. Dong Z. Cui D.. (2017). Genome-wide association study for 13 agronomic traits reveals distribution of superior alleles in bread wheat from the Yellow and Huai Valley of China. Plant Biotechnol. J. 15, 953–969. 10.1111/pbi.12690 28055148 PMC5506658

[B50] Van Ooijen J. (2006). JoinMap^®^ 4, Software for the Calculation of Genetic Linkage Maps in Experimental Populations. Kyazma B.V. Wageningen, Netherlands.

[B51] Wang S. Wong D. Forrest K. Allen A. Chao S. Huang B. E.. (2014). Characterization of polyploid wheat genomic diversity using a high-density 90 000 single nucleotide polymorphism array. Plant Biotechnol. J. 12, 787–796. 10.1111/pbi.12183 24646323 PMC4265271

[B52] Wang Z. Hu H. Jiang X. Tao Y. Lin Y. Wu F.. (2020). Identification and validation of a novel major quantitative trait locus for plant height in common wheat (*Triticum aestivum* L.). Front. Genet. 11:602495. 10.3389/fgene.2020.602495 33193748 PMC7642865

[B53] Wen W. He Z. Gao F. Liu J. Jin H. Zhai S.. (2017). A high-density consensus map of common wheat integrating four mapping populations scanned by the 90K SNP array. Front. Plant Sci. 8:1389. 10.3389/fpls.2017.01389 28848588 PMC5552701

[B54] Wingen L. U. West C. Leverington-Waite M. Collier S. Orford S. Goram R.. (2017). Wheat landrace genome diversity. Genetics 205, 1657–1676. 10.1534/genetics.116.194688 28213475 PMC5378120

[B55] Wu Y. Close T. J. Lonardi S. (2010). Accurate construction of consensus genetic maps via integer linear programming. IEEE/ACM Trans. Comput. Biol. Bioinform. 8, 381–394. 10.1109/TCBB.2010.35 20479505

[B56] Xu Y. Wang R. Tong Y. Zhao H. Xie Q. Liu D.. (2014). Mapping QTLs for yield and nitrogen-related traits in wheat: influence of nitrogen and phosphorus fertilization on QTL expression. Theor. Appl. Genet. 127, 59–72. 10.1007/s00122-013-2201-y 24072207

[B57] Yao J. Zhao D. Chen X. Zhang Y. Wang J. (2018). Use of genomic selection and breeding simulation in cross prediction for improvement of yield and quality in wheat (*Triticum aestivum* L.). Crop J. 6, 353–365. 10.1016/j.cj.2018.05.003

[B58] Yu Y. Ouyang Y. Yao W. (2018). shinyCircos: an R/Shiny application for interactive creation of Circos plot. Bioinformatics 34, 1229–1231. 10.1093/bioinformatics/btx763 29186362

[B59] Zanke C. D. Ling J. Plieske J. Kollers S. Ebmeyer E. Korzun V.. (2014). Whole genome association mapping of plant height in winter wheat (*Triticum aestivum* L.). PLoS ONE 91:e113287. 10.1371/journal.pone.0113287 25405621 PMC4236181

[B60] Zhang L. Li H. Meng L. Wang J. (2020a). Ordering of high-density markers by the k-Optimal algorithm for the traveling-salesman problem. Crop J. 8, 701–712. 10.1016/j.cj.2020.03.005

[B61] Zhang L. Liu P. Wu J. Qiao L. Zhao G. Jia J.. (2020b). Identification of a novel ERF gene, *TaERF8*, associated with plant height and yield in wheat. BMC Plant Biol. 20:263. 10.1186/s12870-020-02473-6 32513101 PMC7282131

[B62] Zhang L. Meng L. Wang J. (2019). Linkage analysis and integrated software GAPL for pure-line populations derived from four-way and eight-way crosses. Crop J. 7, 283–293. 10.1016/j.cj.2018.10.006

[B63] Zhang N. Zhang X. Song L. Su Q. Zhang S. Liu J.. (2020c). Identification and validation of the superior alleles for wheat kernel traits detected by genome-wide association study under different nitrogen environments. Euphytica 216:52. 10.1007/s10681-020-2572-5

[B64] Zhang S. Meng L. Wang J. Zhang L. (2017). Background controlled QTL mapping in pure-line genetic populations derived from four-way crosses. Heredity 119, 256–264. 10.1038/hdy.2017.42 28722705 PMC5597784

