# Supplementary material for: Construction of Consensus Genetic Map With Applications in Gene Mapping of Wheat (Triticum aestivum L.) Using 90K SNP Array
Source: Front Plant Sci. 2021 Aug 25;12:727077. doi: 10.3389/fpls.2021.727077 (PMC8424075; doi:10.3389/fpls.2021.727077)
Supplement: Supplementary file 1 [file Data_Sheet_1.docx]

**1. Supplementary Figures**


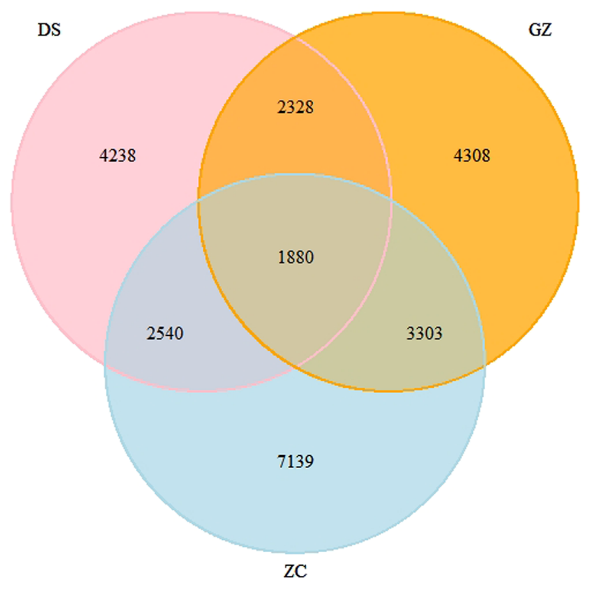


**Supplementary Figure 1.** Numbers of polymorphic SNP markers in three RIL populations Doumai × Shi 4185 (DS), Gaocheng 8901 × Zhoumai 16 (GZ), and Zhou 8425B × Chinese Spring (ZC).


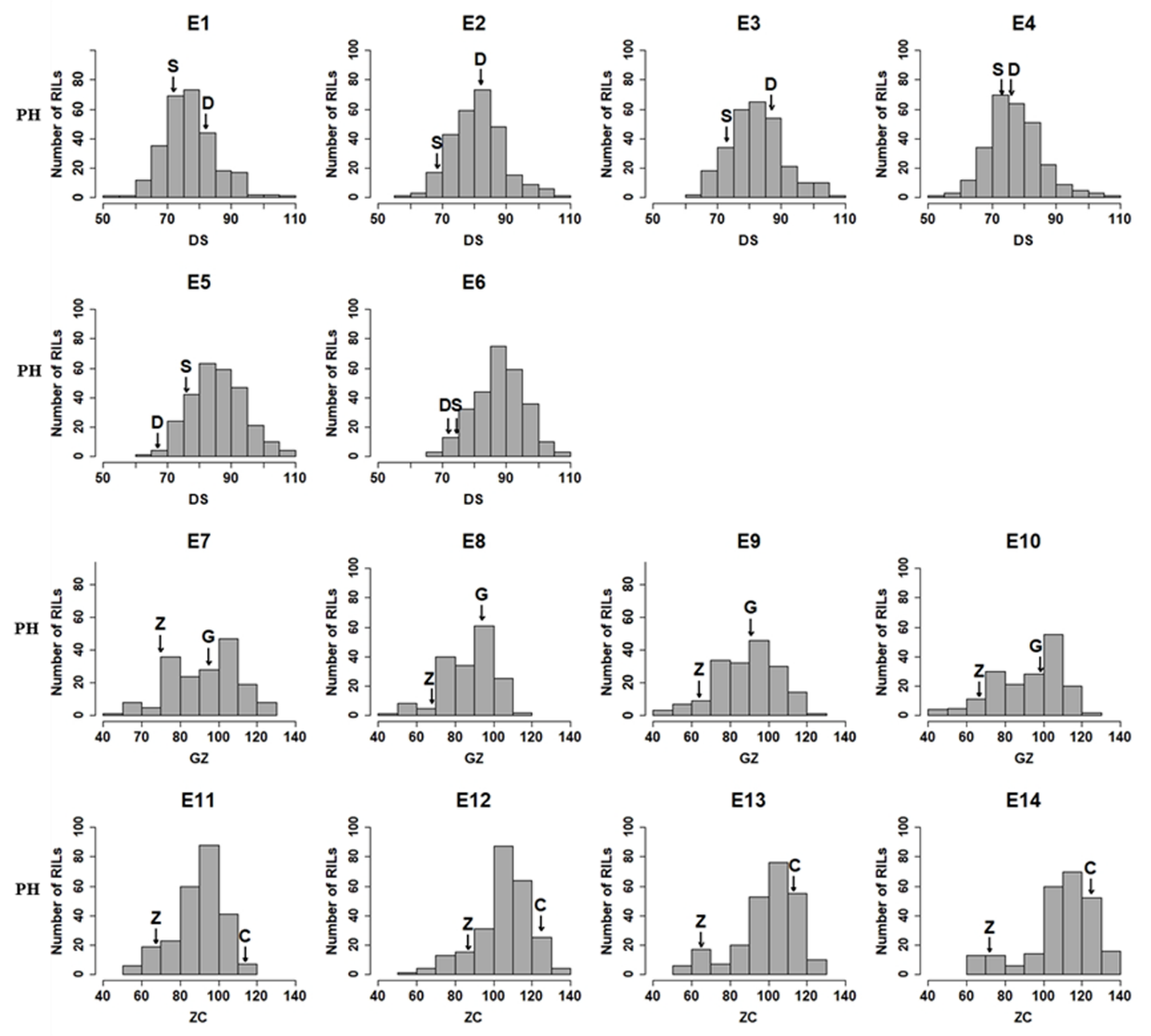


**Supplementary Figure 2.** Frequency distribution of plant height (PH) in three RIL populations Doumai × Shi 4185 (DS), Gaocheng 8901 × Zhoumai 16 (GZ), and Zhou 8425B × Chinese Spring (ZC) grown in a number of environments. E1, 2012-2013 Beijing; E2, 2012-2013 Shijiazhuang; E3, 2013-2014 Beijing; E4, 2013-2014 Shijiazhuang; E5, 2014-2015 Beijing; E6, 2014-2015 Shijiazhuang; E7, 2012-2013 Anyang; E8, 2012-2013 Suixi; E9, 2013-2014 Anyang; E10, 2013-2014 Suixi; E11, Zhoukou2013; E12, Zhengzhou2013; E13, Zhoukou2014; E14, Zhengzhou2014.


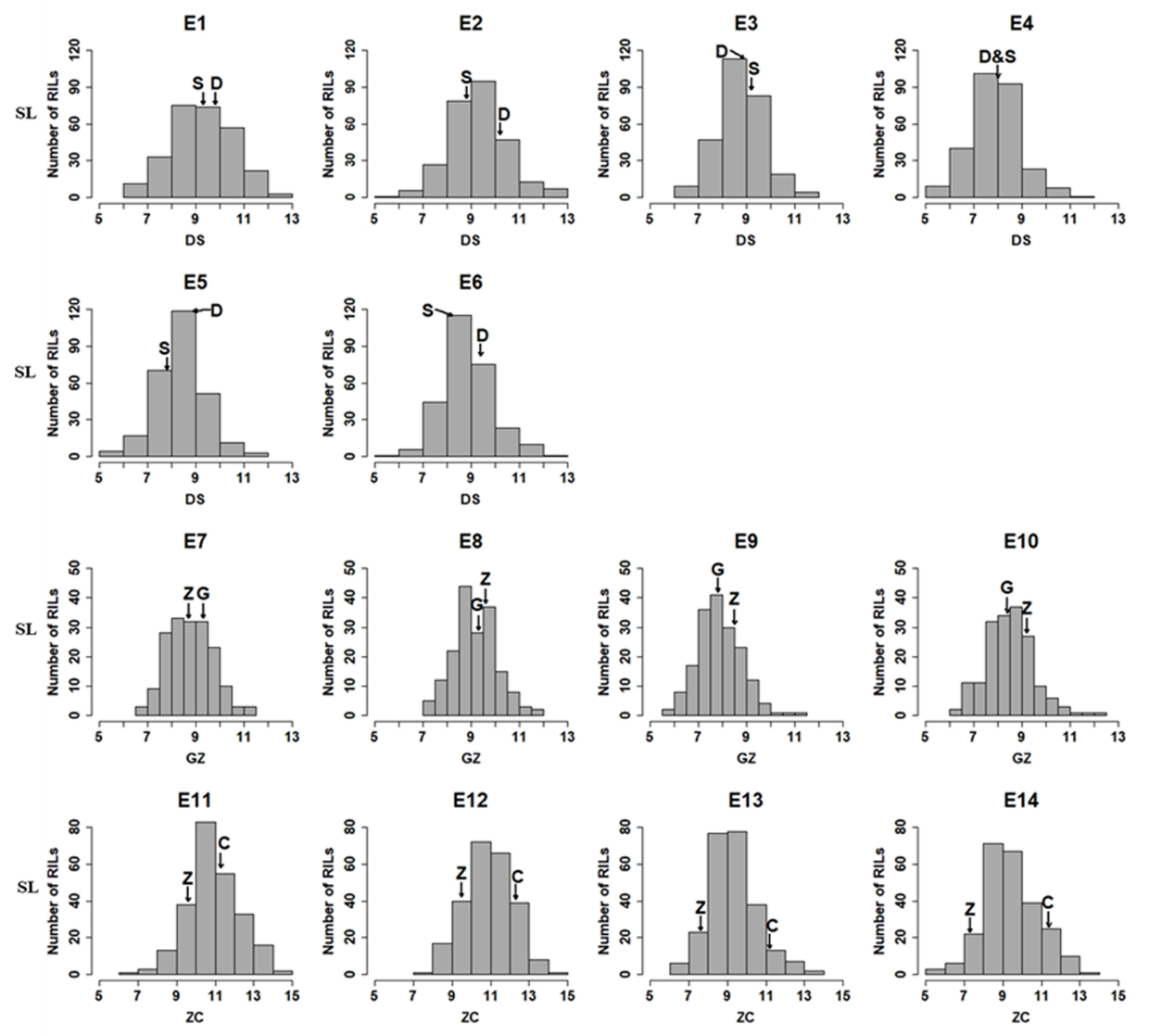


**Supplementary Figure 3.** Frequency distribution of spike length (SL) in three RIL populations Doumai × Shi 4185 (DS), Gaocheng 8901 × Zhoumai 16 (GZ), and Zhou 8425B × Chinese Spring (ZC) grown in a number of environments. E1, 2012-2013 Beijing; E2, 2012-2013 Shijiazhuang; E3, 2013-2014 Beijing; E4, 2013-2014 Shijiazhuang; E5, 2014-2015 Beijing; E6, 2014-2015 Shijiazhuang; E7, 2012-2013 Anyang; E8, 2012-2013 Suixi; E9, 2013-2014 Anyang; E10, 2013-2014 Suixi; E11, Zhoukou2013; E12, Zhengzhou2013; E13, Zhoukou2014; E14, Zhengzhou2014.


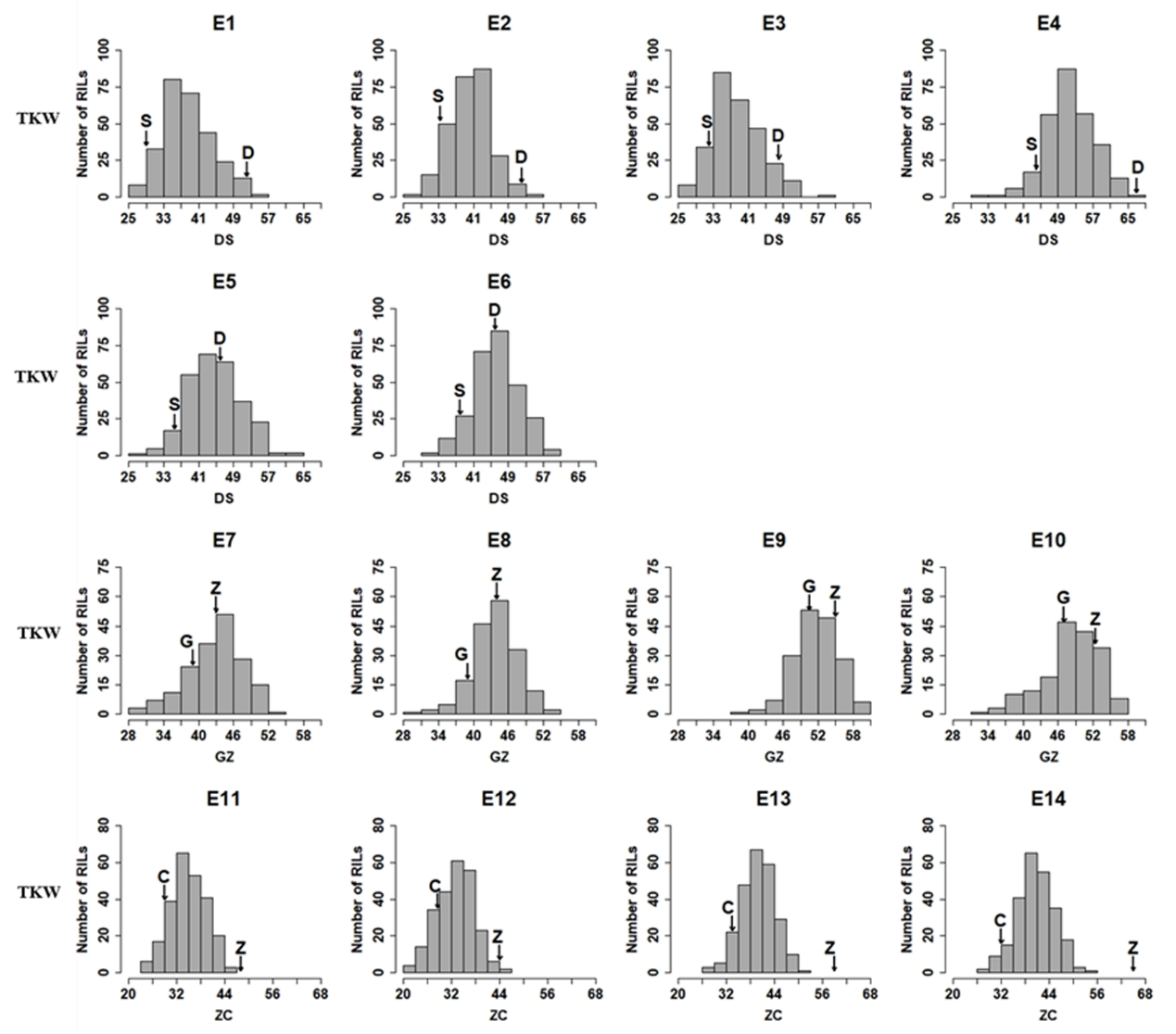


**Supplementary Figure 4.** Frequency distribution of thousand-kernel weight (TKW) in three RIL populations Doumai × Shi 4185 (DS), Gaocheng 8901 × Zhoumai 16 (GZ), and Zhou 8425B × Chinese Spring (ZC) grown in a number of environments. E1, 2012-2013 Beijing; E2, 2012-2013 Shijiazhuang; E3, 2013-2014 Beijing; E4, 2013-2014 Shijiazhuang; E5, 2014-2015 Beijing; E6, 2014-2015 Shijiazhuang; E7, 2012-2013 Anyang; E8, 2012-2013 Suixi; E9, 2013-2014 Anyang; E10, 2013-2014 Suixi; E11, Zhoukou2013; E12, Zhengzhou2013; E13, Zhoukou2014; E14, Zhengzhou2014.


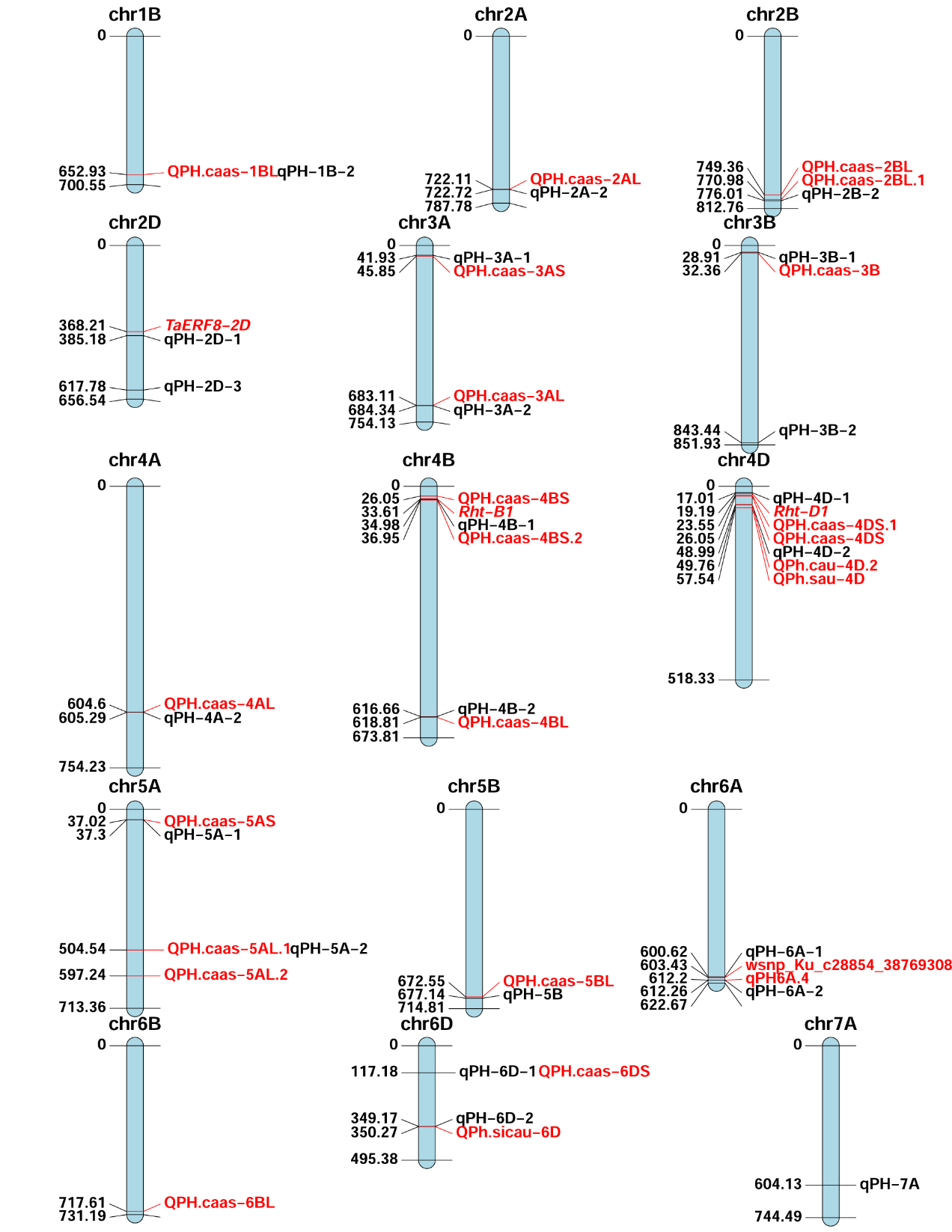


**Supplementary Figure 5.** Physical map of QTLs identified for plant height (PH). Stable QTLs detected based on consensus map are highlighted in black with their respective physical positions (in Mb), while previously reported QTLs/markers are labeled in red.


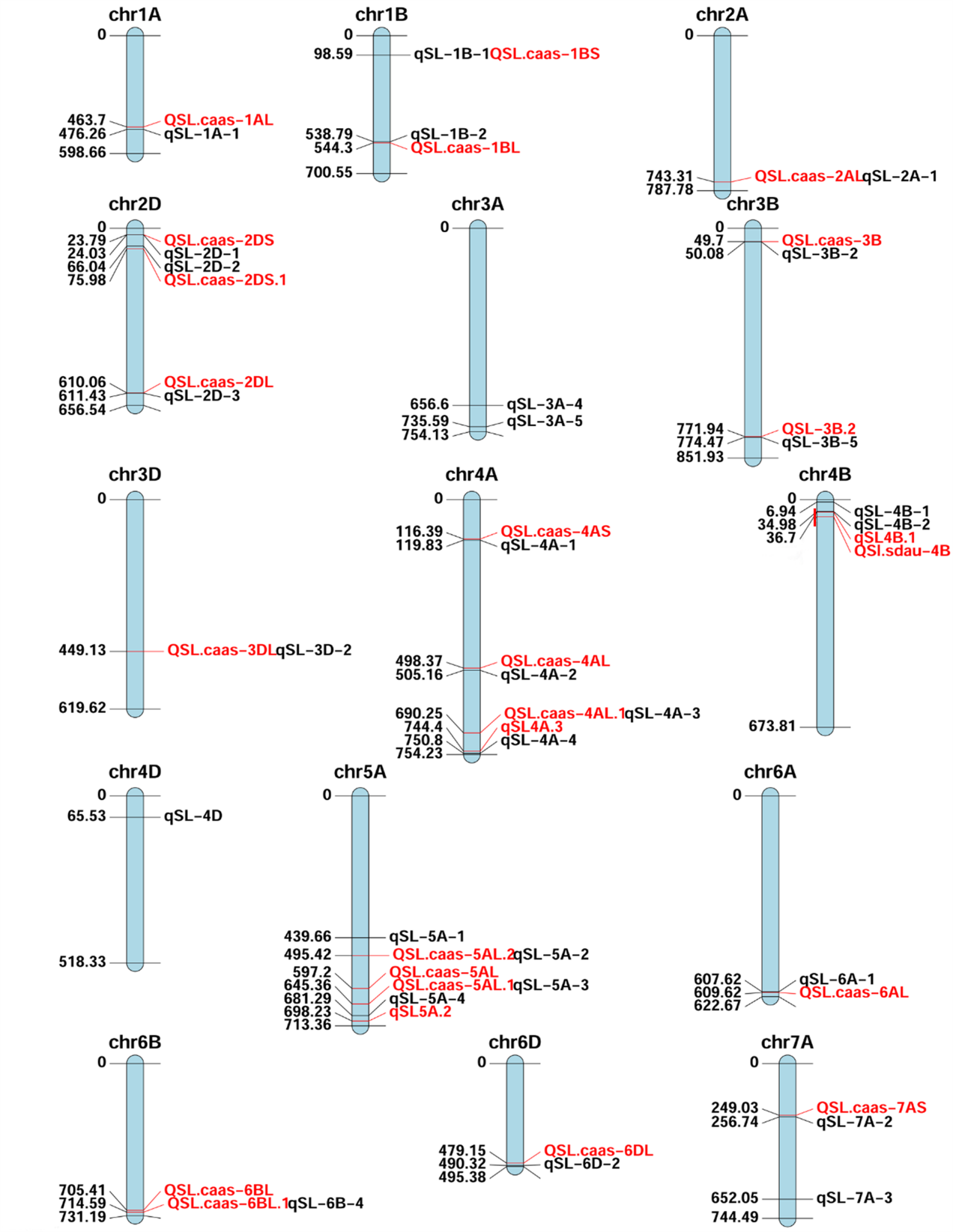


**Supplementary Figure 6.** Physical map of QTLs identified for spike length (SL). Stable QTLs detected based on consensus map are highlighted in black with their respective physical positions (in Mb), while previously reported QTLs/markers are labeled in red.

**
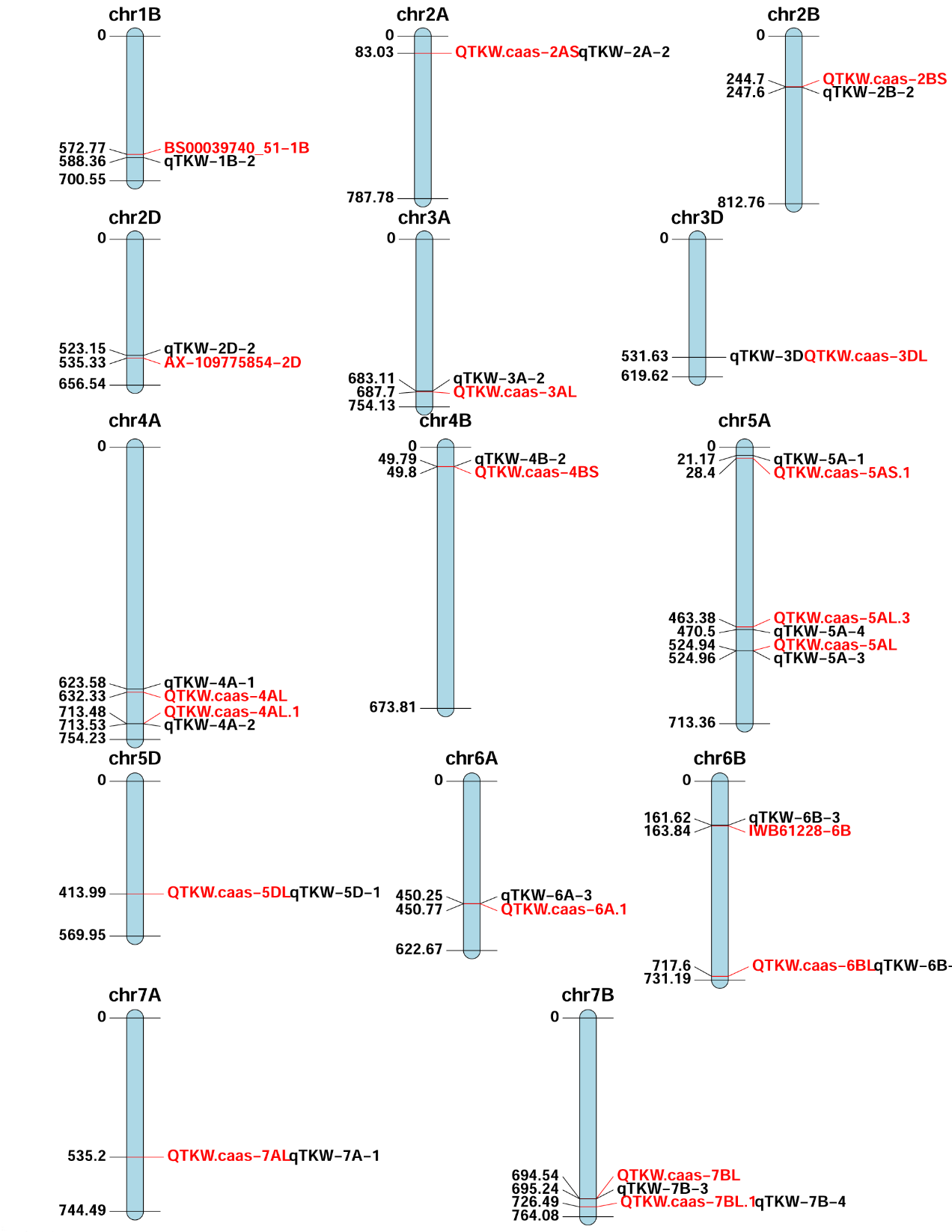
**

**Supplementary Figure 7.** Physical map of QTLs identified for thousand-kernel weight (TKW). Stable QTLs detected based on consensus map are highlighted in black with their respective physical positions (in Mb), while previously reported QTLs/markers are labeled in red.
